# Integrative Lipidomics Reveals *Schizochytrium* Oil-Induced Muscle Lipid Remodeling Associated with Meat Quality, Oxidative Stability, and Flavor in *Hu* Lambs

**DOI:** 10.3390/foods15203753

**Published:** 2026-10-21

**Authors:** Rongyu Yao, Haiyu Ma, Danian Chen, Xiaoli Zhou, Peidi Zhao, Xiuxiu Weng, Wanhong Li

**Affiliations:** 1 State Key Laboratory of Herbage Improvement and Grassland Agro-Ecosystems, College of Pastoral Agriculture Science and Technology, Lanzhou University, Lanzhou 730020, China; 120220900691@lzu.edu.cn (R.Y.); mahy@dxvtc.edu.cn (H.M.); wyzhaopd@163.com (P.Z.); wengxx@lzu.edu.cn (X.W.); 2 College of Veterinary and Animal Science, Dingxi Vocational and Technical College, Dingxi 730500, China; 3 Dongxiang County Mutton Sheep Industry Research Center, Dongxiang County, Linxia 731400, China; cdn1767480328@163.com (D.C.); zhouxiaoli0106@163.com (X.Z.)

**Keywords:** *Schizochytrium* oil, lipid remodeling, lipidomics, oxidative stability, flavor

## Abstract

Intensive grain feeding in lamb meat production can promote excessive n-6 polyunsaturated fatty acid (PUFA) deposition, which may compromise meat quality and flavor. This study investigated the response of *Hu* lamb meat to dietary docosahexaenoic acid (DHA)-rich *Schizochytrium* oil (SO), with particular attention paid to meat quality, antioxidant status, muscle lipids, and flavor. SO supplementation significantly enhanced meat lightness, activities of antioxidant enzymes, expression of antioxidant genes, DHA content, and the n-3/n-6 PUFA ratio. Lipidomic profiling detected 447 differential lipid metabolites (DLMs) between the two groups. The SO group showed higher levels of n-3 PUFAs, including docosahexaenoic acid (DHA) and eicosapentaenoic acid (EPA), but lower levels of n-6 PUFAs, including C22:4. A flavoromic analysis identified 197 differential VOCs and showed enhanced green, fatty, and fruity aroma attributes in the SO group. Spearman correlation analysis and the random forest model were used to identify DLMs associated with differential meat quality traits, differential antioxidant enzyme activities, and key Volatile organic compounds. Three DLMs were identified as candidate lipid biomarkers, including PE(O-18:2/22:6), LPE(P-16:1), and PE(O-16:2/22:6). The results indicate that the remodeling of muscle lipids induced by SO could play a role in enhancing the quality and flavor attributes of lamb meat.

## 1. Introduction

Sheep meat is an important source of high-quality animal protein worldwide, and the increasing demand for mutton has promoted the development of intensive feeding systems to improve production efficiency. However, the transition from traditional grazing to grain-based feeding has substantially altered muscle lipid metabolism, resulting in the excessive deposition of n-6 polyunsaturated fatty acids (PUFAs) and an imbalance in the n-3/n-6 PUFA ratio [1]. This imbalance not only reduces the nutritional value of lamb meat but also affects the lipid oxidation, flavor formation, and physicochemical properties, thereby influencing consumer acceptance [2]. Therefore, strategies aimed at improving the muscle fatty acid profile and optimizing the lipid metabolism are essential for enhancing the lamb meat quality.

Lipids are not merely energy storage molecules but also critical regulators of meat quality characteristics. Different lipid subclasses exhibit distinct biological functions due to their structural composition and susceptibility to oxidation. Phospholipids (PLs), which contain abundant PUFAs, are highly prone to oxidative degradation and serve as important precursors for volatile compounds contributing to meat flavor development [3]. For example, aldehydes such as hexanal, which are key contributors to roasted lamb flavor, are mainly generated through the oxidation of PUFA-containing phospholipids [4]. In contrast, triacylglycerols (TGs), as the predominant components of intramuscular fat (IMF), contribute to meat tenderness by improving the lubrication between muscle fibers and reducing the protein density [5]. Importantly, the functional properties of lipids are determined not only by their total abundance but also by the specific fatty acids incorporated into individual lipid molecular species [6,7]. Thus, understanding lipid remodeling at the subclass and molecular species levels is critical for elucidating the mechanisms underlying meat quality regulation.

Dietary strategies can modify the muscle lipid composition by promoting the incorporation of beneficial n-3 PUFA. Because mammals synthesize long-chain n-3 PUFA only to a limited extent, increasing their levels in tissues largely depends on the dietary supply. Among n-3 PUFAs, docosahexaenoic acid (DHA) exhibits stronger biological activities than other fatty acids and plays important roles in regulating inflammation, oxidative balance, and lipid metabolism [8]. In addition, DHA may also alter the rumen microbiota community structure and reduce ruminal biohydrogenation, which may reduce the loss of unsaturated fatty acids [9]. *Schizochytrium* oil, which is a microbial oil extracted from fermented *Schizochytrium* sp., is rich in DHA and has emerged as a promising alternative source of n-3 PUFAs. Compared with fish oil, *Schizochytrium* oil offers several benefits, such as a high content of DHA, a stable composition, and natural antioxidants like carotenoids and vitamin E, which could enhance oxidative stability [10]. Earlier studies in ruminants have shown that dietary *Schizochytrium* oil supplementation can increase DHA deposition in ruminant muscle and improve certain meat quality characteristics, including a decreased shear force and reduced drip loss [11,12].

However, previous studies have mainly concentrated on alterations in the overall composition of fatty acids, which cannot fully characterize the complex lipid remodeling processes occurring in muscle. The specific alterations in lipid subclasses and molecular species induced by *Schizochytrium* oil, as well as their relationships with meat quality traits, oxidative stability, and flavor formation, remain largely unknown. Therefore, a comprehensive understanding of lipid remodeling mechanisms is required to identify candidate lipid molecules involved in quality regulation.

This study aims to characterize the lipid remodeling associated with the quality improvement of lamb fed *Schizochytrium* oil. By integrating lipidomics, GC-MS, antioxidant assays, and gene expression analysis, we constructed a network linking specific lipid species to meat quality traits and examined the associations of these lipid alterations with meat quality, antioxidant capacity, and flavor profiles. Ultimately, this work provides new insights into the lipid remodeling associated with lamb meat quality and identifies candidate lipid biomarkers (CLBs) for evaluating SO-induced changes in meat quality.

## 2. Methods

### 2.1. Animals, Diets, and Management Strategy

The study was conducted at Minqin Defu Sheep Industry Co., Ltd., Wuwei, Gansu Province, China. The Animal Ethics and Welfare Committee of the Institute of Ruminant Research at Lanzhou University approved all experimental protocols (approval number: CY20240712). Twenty healthy male *Hu* lambs with similar body weights and ages (initial body weight 21.84 ± 0.47 kg; age 3.0 ± 0.1 months) were randomly divided into two groups, each consisting of 10 lambs. The lambs were maintained under uniform environmental conditions. Feed was provided at 09:00 and 17:00 each day, with fresh water provided ad libitum throughout the trial. After a 7-day acclimatization period, an 84-day feeding trial was conducted.

The diets for the experiments were created based on guidelines provided by the National Research Council [13] and prepared as total mixed pelleted diets (Table S1). In the control (CK) group, the basal diet was provided, while the SO group was given the same diet with 1% of palm oil substituted with an equivalent volume of SO. The SO was sourced from Shandong Naichen Biotechnology Co., Ltd. (Jinan, China). According to compositional analysis conducted by Pony Testing International Group (Qingdao, China), the SO contained 49.7% DHA, 0.6% eicosapentaenoic acid (EPA), 51.0% total n-3 PUFAs, and 0.4% total n-6 PUFAs.

### 2.2. Feed Composition Analysis

Feed samples were analyzed for nutrient composition according to AOACmethods [14]. After drying at 65 °C for 2 days, ash, acid detergent fiber (ADF), neutral detergent fiber (NDF), crude fat (CF), crude protein (CP), and dry matter (DM) were determined. The composition of fatty acids was assessed following the methodology outlined by Zhang et al. [15]. Table S1 displays the nutritional content and fatty acid profiles of the diets.

### 2.3. Sample Collection

Blood samples were retrieved from the jugular vein 24 h before slaughter. These samples were maintained at 25 °C for one hour and subsequently centrifuged at 3000× *g* for 15 min. The serum was collected and stored at −80 °C. All lambs were allowed unrestricted access to water, and, after fasting for 16 h, slaughtered following Chinese local standard DB13/T 963-2008 as procedure by Zhang et al. [16]. Immediately post-slaughter, *longissimus dorsi* muscle samples were collected between the 12th and 13th ribs. For a histological examination, samples were preserved in a 4% paraformaldehyde solution (Wuhan Servicebio Technology Co., Ltd., Wuhan, China). Samples for fatty acid analysis, antioxidant analysis, VOC analysis, lipidomics, and qRT-PCR were stored at −80 °C. A segment of the *longissimus dorsi* muscle was analyzed for meat quality.

### 2.4. Muscle Microstructure Analysis

Fixed *longissimus dorsi* samples were processed for paraffin embedding and sectioned at 5 μm as described by Zhang et al. [15]. The sections were stained with hematoxylin and eosin and observed under a microscope. Representative histological images were acquired at 200× magnification, whereas three fields from each sample were examined at 100× magnification for quantitative analysis. Measurements of muscle perimysium thickness, circumference, diameter, and muscle fiber cross-sectional area were performed using cellSens Standard software (Olympus, Tokyo, Japan).

### 2.5. Meat Quality Analysis

The pH and color of the *longissimus dorsi* muscle were measured 45 min post-slaughter. Each sample was measured at three different points, and the mean value was used for analysis. Meat color was measured using a Minolta chromameter (Konica Minolta Sensing Inc., Osaka, Japan) equipped with an 8 mm aperture, D65 light source, and a 2-degree standard, and was expressed as L* (lightness), a* (redness), and b* (yellowness). The pH value was determined using a pH meter (HANNA Instruments Inc., Woonsocket, RI, USA; HI99163). After pH and color were measured, the meat samples were stored at 4 °C and 90% relative humidity for 24 h. The press loss, cooking loss, shear force, drip loss, and texture characteristics were then evaluated, and pH and color were measured again at the same locations. For press loss, approximately 2 g of each sample was positioned between 18 pieces of qualitative filter paper and compressed under 35 kg for 5 min using an RH-1000 meat press (Runhu Instrument Co., Ltd., Guangzhou, China). Press loss was calculated as [(W_1_ − W_2_)/W_1_] × 100, where W_1_ and W_2_ represent the sample weights before and after pressing, respectively. For cooking loss, approximately 10 g of each sample was weighed and steamed for 30 min, then cooled to room temperature, blotted dry, and weighed again, with the change in sample weight used to calculate cooking loss. For shear force measurement, approximately 50 g of each meat sample was placed in a water bath maintained at 80 °C until the internal temperature reached 70 °C. Ten cylindrical cores (1 cm in diameter) were then taken from each sample parallel to the muscle fiber direction. Shear force was determined with a C-LM3 meat tenderness meter (Bulader Technology Development Co., Ltd., Beijing, China) at a vertical speed of 5 mm/s, recording the average force from 10 measurements. For drip loss, three samples were placed into a sealed inflated plastic bag, which was then kept at 4 °C for an additional 24 h. Sample weight loss during this period was used to determine drip loss. Furthermore, a texture profile analysis was conducted utilizing a TMS-Pro texture analyzer (Food Technology Corporation, Sterling, VA, USA).

Fatty acid composition was determined as described by Zhang et al. [15]. In summary, lipids were extracted from 0.2 g of a freeze-dried sample with a 2:1 (*v*/*v*) mixture of chloroform and methanol. Following extraction, the lipids underwent methylation with 2 mL of sodium methoxide. To obtain fatty acid methyl esters (FAMEs), after methylation, 2 mL each of distilled water and hexane-containing methyl heneicosanoate (Sigma-Aldrich, St. Louis, MO, USA) were added to the methylation mixture. The mixture was vortexed for 5 min and centrifuged at 800× *g* for 10 min. Subsequently, 1 mL of the upper organic phase was carefully collected and transferred to a GC vial for analysis. FAMEs were conducted by gas chromatograph (Thermo Fisher Scientific, Waltham, MA, USA) outfitted with flame ionization detector using an SP-2560 fused silica capillary column (100 m × 0.25 mm × 0.2 μm, Sigma-Aldrich, St. Louis, MO, USA). The temperature protocol was outlined as follows: starting at 130 °C for 1 min, increasing to 170 °C at 6.5 °C/min, then progressing to 215 °C at 2.75 °C/min, maintained for 12 min, ramped to 230 °C at 4 °C/min, and held for an extra 3 min. The temperature of the detector was consistently kept at 280 °C. Supelco 37 Component FAME Mix (Supelco, Sigma-Aldrich, St. Louis, MO, USA, CRM47885) served as FAME standards for identifying the fatty acid profile of the samples. Sample peaks were recognized according to retention time, and the concentrations of the individual fatty acids were calculated by contrasting the peak areas with those of the known standards.

### 2.6. Antioxidant Capacity Analysis

Tissue homogenates were prepared from approximately 100 mg of *longissimus dorsi* muscle powder mixed with 900 μL of PBS, followed by homogenization and centrifugation. The mixture was centrifuged at 3000× *g* for 10 min at 4 °C. The supernatant was collected for the subsequent measurement of antioxidant enzyme activities. The protein concentrations in the final supernatant were determined using the BCA assay. The serum and muscle antioxidant status was evaluated with commercial kits from Nanjing Jiancheng Bioengineering Institute (Nanjing, China), following the supplier’s protocols [17]. The measurements included superoxide dismutase (SOD), catalase (CAT), and glutathione peroxidase (GSH-Px) activities, total antioxidant capacity (T-AOC), and malondialdehyde (MDA) levels.

### 2.7. Flavoromics Analysis

Volatile organic compounds (VOCs) in the *longissimus dorsi* muscle were analyzed by headspace solid-phase microextraction coupled with gas chromatography–mass spectrometry (HS-SPME-GC-MS). The 200 mg sample ground in liquid nitrogen was placed in a 20 mL headspace vial with 200 mg NaCl and 20 μL of 3-Hexanone-2,2,4,4-d4 (10 μg/mL). After equilibration for 5 min at 60 °C, headspace VOCs were collected for 15 min at the same temperature using a 120 μm DVB/CWR/PDMS SPME Arrow (Agilent Technologies, Santa Clara, CA, USA). The SPME Arrow was then transferred to the GC inlet for thermal desorption at 250 °C for 5 min under splitless conditions. GC-MS analysis was performed using an Agilent 8890 GC coupled to a 7000D mass spectrometer (Agilent Technologies, Santa Clara, CA, USA) with a DB-5MS capillary column (30 m × 0.25 mm × 0.25 μm; Agilent J&W Scientific, Folsom, CA, USA). The carrier gas was high-purity helium, delivered at 1.2 mL/min. The GC oven was initially set at 40 °C for 3.5 min and then increased to 100 °C at 10 °C/min, 180 °C at 7 °C/min, and 280 °C at 25 °C/min, with a final hold of 5 min.

Mass spectra were obtained using electron ionization mode at a voltage of 70 eV. The transfer line was maintained at 280 °C. The ion source and quadrupole were kept at 230 and 150 °C, respectively, and mass spectral data were collected in selected ion monitoring mode for VOC identification and quantification. VOCs identification relied on an in-house database that encompassed data from various species, published studies, available authentic standards, and retention index information. This database contained reference retention times and characteristic ions for the target VOCs. For each compound, a quantifier ion along with two to three qualifier ions were chosen. Identification of a VOC occurred when its retention time corresponded with the reference value and the characteristic ions were detected following background subtraction. Raw data processing was carried out using MassHunter software for the purposes of peak integration and correction. Relative VOC contents were determined through a semi-quantitative method based on an internal standard. For each VOC, the chromatographic response was normalized to that of the internal standard and adjusted according to the amount of standard added and sample mass.

Missing values were replaced with one-fifth of the minimum value for each VOC, and VOCs showing a coefficient of variation < 0.5 across quality-control samples were retained for further analysis. Differential VOCs were screened using VIP > 1, *p* < 0.05, and |log_2_FC| > 1. The *p* values were calculated using a two-sided Welch’s *t*-test. The relative odor activity value (rOAV) was defined as rOAV_i_ = C_i_/T_i_, where C_i_ is the relative content of VOC_i_ and T_i_ is its odor threshold obtained from published literature. Differential VOCs with rOAV > 1 were defined as key VOCs.

### 2.8. Lipidomics Analysis

Muscle lipids were extracted using a modified MTBE-based procedure adapted from Gao et al. [12]. Briefly, frozen muscle samples were ground in liquid nitrogen. For each sample, 20 mg of tissue powder was extracted with 1 mL of a solvent containing 75% methyl tert-butyl ether and 25% methanol (*v*/*v*) together with the internal standard mixture, followed by vortexing for 15 min. Then, 200 μL of ultrapure water was added to induce phase separation. After a further 1 min of mixing, the resulting mixture was centrifuged at 12,000 rpm for 10 min. The upper organic phase (200 μL) was collected into a fresh tube and evaporated to dryness under vacuum. The dried extract was reconstituted in 200 μL of an equal volume mixture of acetonitrile and isopropanol for lipidomic analysis.

Lipidomic profiling employed an ExionLC AD UPLC system coupled to a QTRAP 6500+ mass spectrometer (SCIEX, Framingham, MA, USA). An Accucore C30 column (2.6 μm, 2.1 mm × 100 mm; Thermo Fisher Scientific, Waltham, MA, USA) facilitated the separation of lipids. Solvent A consisted of acetonitrile and water at a ratio of 6:4 (*v*/*v*) and contained 0.1% formic acid and 0.01 mol/L ammonium formate, whereas solvent B contained acetonitrile and isopropanol at a ratio of 1:9 (*v*/*v*), with the same additives. As solvents A and B totaled 100%, the gradient was expressed as the proportion of solvent B. Solvent B was set at 20% at 0 min, 30% at 2 min, 60% at 4 min, 85% at 9 min, 90% at 14 min, and 95% from 15.5 to 17.3 min. It was then reduced to 20% and maintained until 20 min. Each sample was injected at a volume of 2 μL, while the column was kept at 45 °C and the mobile phase was delivered at 350 μL/min.

Electrospray ionization was performed in both positive and negative modes. The source temperature was 500 °C, with spray potentials of +5.5 kV in positive mode and −4.5 kV in negative mode. Gas pressures were 45 psi for GS1, 55 psi for GS2, and 35 psi for the curtain gas. Lipids were identified using the in-house lipid database based on retention time and precursor and product ion pairs. Lipid contents were measured by multiple reaction monitoring. Nitrogen was used as the collision gas at 5 psi, and the declustering potential along with the collision energy were fine-tuned for each transition. Lipid contents were calculated from the peak area ratio between each lipid and the internal standard, together with the internal standard concentration, correction factor, extraction volume, and sample weight, and were expressed as nmol/g. The raw data from the mass spectrometry were analyzed using Analyst 1.6.3 software. Differential lipid metabolites (DLMs) were screened using VIP > 1 and *p* < 0.05. The p values were calculated using a two-sided Welch’s *t*-test.

### 2.9. RNA Isolation, cDNA Synthesis and qRT-PCR Analysis

RNA isolation, cDNA synthesis, and qRT-PCR analysis were performed as described by Yao et al. [18]. In summary, total RNA was isolated using the RNAprep Pure Tissue Kit (TIANGEN Biotech Co., Ltd., Beijing, China) according to the manufacturer’s instructions. RNA concentration and purity were assessed with NanoDrop 2000 (Thermo Fisher Scientific, Waltham, MA, USA), and integrity was checked by agarose gel electrophoresis. Only RNA samples within the specified purity ranges (2.0 < OD260/230 < 3.0 and 1.9 < OD260/280 < 2.1) were adjusted to 500 ng/µL and reverse-transcribed using EasyScript^®^ One-Step gDNA Removal and cDNA Synthesis SuperMix (TransGen Biotech, Beijing, China). The resulting cDNA was diluted with nuclease-free water at a 1:4 ratio for subsequent gene expression analysis.

qRT-PCR was performed using a CFX96 Real-Time System (Bio-Rad, Hercules, CA, USA) with TransStart^®^ Tip Green qPCR SuperMix (TransGen Biotech, Beijing, China). The reaction mixture consisted of 10 µL of 2 × TransStart^®^ Tip Green qPCR SuperMix, 1 µL of cDNA, and 1 µL each of forward and reverse primers (10 µM), with nuclease-free water up to 20 µL. The amplification conditions were set as follows: an initial preheating step at 94 °C for 3 min, followed by 40 cycles at 94 °C for 5 s and 60 °C for 30 s, culminating in a melting curve analysis. *GAPDH* served as the reference gene in this study. The relative expression of genes was calculated using the 2^−ΔΔCt^ method, with all primers detailed in Table S2.

### 2.10. Machine Learning

All analyses were performed in Python v3.9.13 using the pandas v2.3.1, scikit-learn v1.6.1, and XGBoost libraries v2.1.4. Datasets comprising differential meat quality traits, differential antioxidant enzyme activities, and key VOCs, and differential lipid metabolites (DLMs) were merged by sample ID. Variables exhibiting over 50% of missing values were excluded, while the left-out missing data were handled through the application of the K-nearest neighbors algorithm. All predictor variables were standardized by Z-score normalization. Because the all response variables were continuous, the analysis was treated as a regression-type prediction task. Five regression algorithms, including Bayesian Ridge Regression, Elastic Net Regression, Random Forest Regression, Support Vector Regression (SVR), and XGBoost Regression, were used to build predictive models exploring the relationships between DLMs and meat quality-related traits. These models were selected to cover different regression methods used in metabolomics [19]. Bayesian Ridge and Elastic Net were used as regularized linear models, while Random Forest and XGBoost were used as tree-based models and SVR as a kernel-based model. Elastic Net is also useful when predictors are numerous and correlated [20]. Similar regression approaches have been used in metabolomics and meat quality prediction [21,22]. Each model was trained and tested over 100 iterations using an 80/20 random split strategy. Model performance was evaluated using mean absolute error (MAE) and root mean square error (RMSE). For each model, MAE and RMSE were summarized as the mean and standard deviation across the 100 repeated train/test splits, and the 2.5th and 97.5th percentiles were calculated as empirical intervals to characterize variation in model performance across data splits. A performance score was defined as follows:

$$\mathrm{performance}\_\mathrm{score}=\frac{1}{{\mathrm{avg}}_{\mathrm{MAE}}+{\mathrm{avg}}_{\mathrm{RMSE}}+1\times 10^{-8}}$$

In this formula, *avg_MAE_* and *avg_RMSE_* represent the average MAE and RMSE across 100 iterations, respectively. The constant 1 × 10^−8^ was added to avoid division by zero. For each phenotype, the five regression models were ranked according to their performance scores, and the model most frequently ranked first across phenotypes was selected for subsequent feature selection. The distribution of model ranks across phenotypes was further summarized in Supplementary Figure S1. The selected model was then used for recursive feature elimination with cross-validation (RFECV), with five-fold cross-validation, a step size of 1, and a minimum of five retained features. Following feature selection, the model was retrained using the selected subset of metabolites.

### 2.11. Statistical Analysis

The data are expressed as mean ± standard error of the mean (SEM). The Shapiro–Wilk and Levene tests were used to examine normality and equality of variances, respectively. Group differences were analyzed in R v4.4.1 using independent-samples Student’s *t*-tests (normality and equal variances), Welch’s *t*-tests (normality and unequal variances), or Mann–Whitney U tests (non-normal data). The value of *p* < 0.05 was considered statistically significant. Key VOCs were selected from differential VOCs based on rOAV > 1. Spearman correlation coefficients between differential meat quality traits, differential antioxidant enzyme activities, key VOCs, and DLMs were calculated using the Hmisc package v4.40 in R v4.6.1. Significant correlations were defined as |r| > 0.7 and *p* < 0.05. Correlation networks were visualized using Gephi v0.9.3, and bar graphs were plotted with GraphPad Prism v8.0.

## 3. Results

### 3.1. Growth Performance

Table 1 presents the growth performance results of *Hu* lambs fed CK or SO diets. No significant differences (*p* > 0.05) were observed in initial body weight, final body weight, or body weights at each time point throughout the feeding period up to 84 days. The average daily gain (ADG) of the *Hu* lambs in the SO group was significantly higher than that of the CK group from day 57 to day 84 (*p* < 0.01), and the overall ADG also showed an increased tendency (*p* = 0.06).

### 3.2. Meat Quality

Table 2 illustrates the meat quality results of *Hu* lambs fed CK or SO diets. In contrast to the CK group, a notable enhancement in L* at 45 min, L* at 24 h, and gumminess was observed in the SO group (*p* < 0.05). However, the SO group had a significantly lower springiness (*p* < 0.05).

### 3.3. Muscle Fiber Microstructure

**Figure 1:**
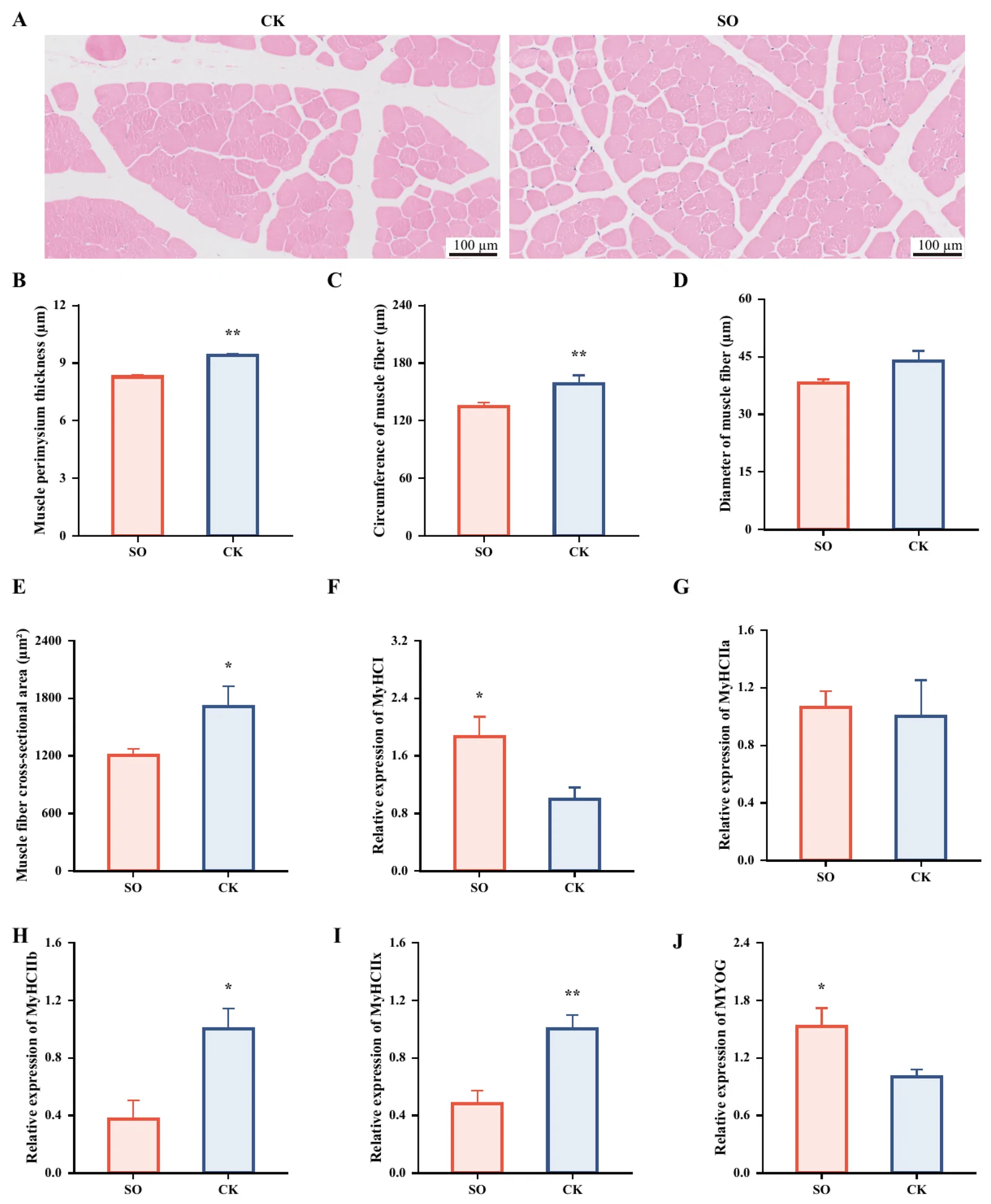
Muscle fiber micromorphology, quantitative traits, and fiber-type-related gene expression in the *longissimus dorsi* muscle of *Hu* lambs fed the SO or CK diets: (**A**) the micromorphology of muscle fiber (magnification, 200×, bar = 100 μm); (**B**) muscle perimysium thickness; (**C**) circumference of muscle fiber; (**D**) diameter of muscle fiber; (**E**) muscle fiber cross-sectional area; and (**F**–**J**) the relative mRNA expression levels of muscle fiber-related genes, including *MyHC I*, *MyHC IIa*, *MyHC IIb*, *MyHC IIx*, and *MYOG*. Values are presented as the means ± SEM. * *p* < 0.05, ** *p* < 0.01.

Figure 1 illustrates the microstructure of muscle fiber in *Hu* lambs fed SO or CK diets. Figure 1A presents the histological sections of muscle fiber. The SO group showed a significant reduction in muscle perimysium thickness, circumference, and muscle fiber cross-sectional area relative to the CK group (*p* < 0.05), while muscle fiber diameter tended to decrease (*p =* 0.08; Figure 1B–E). In the SO group, *MyHC I* and *MYOG* expression were upregulated, whereas *MyHC IIb* and *MyHC IIx* expression exhibited the opposite trend (*p* < 0.05, Figure 1F–J).

### 3.4. Antioxidant Capacity

**Figure 2:**
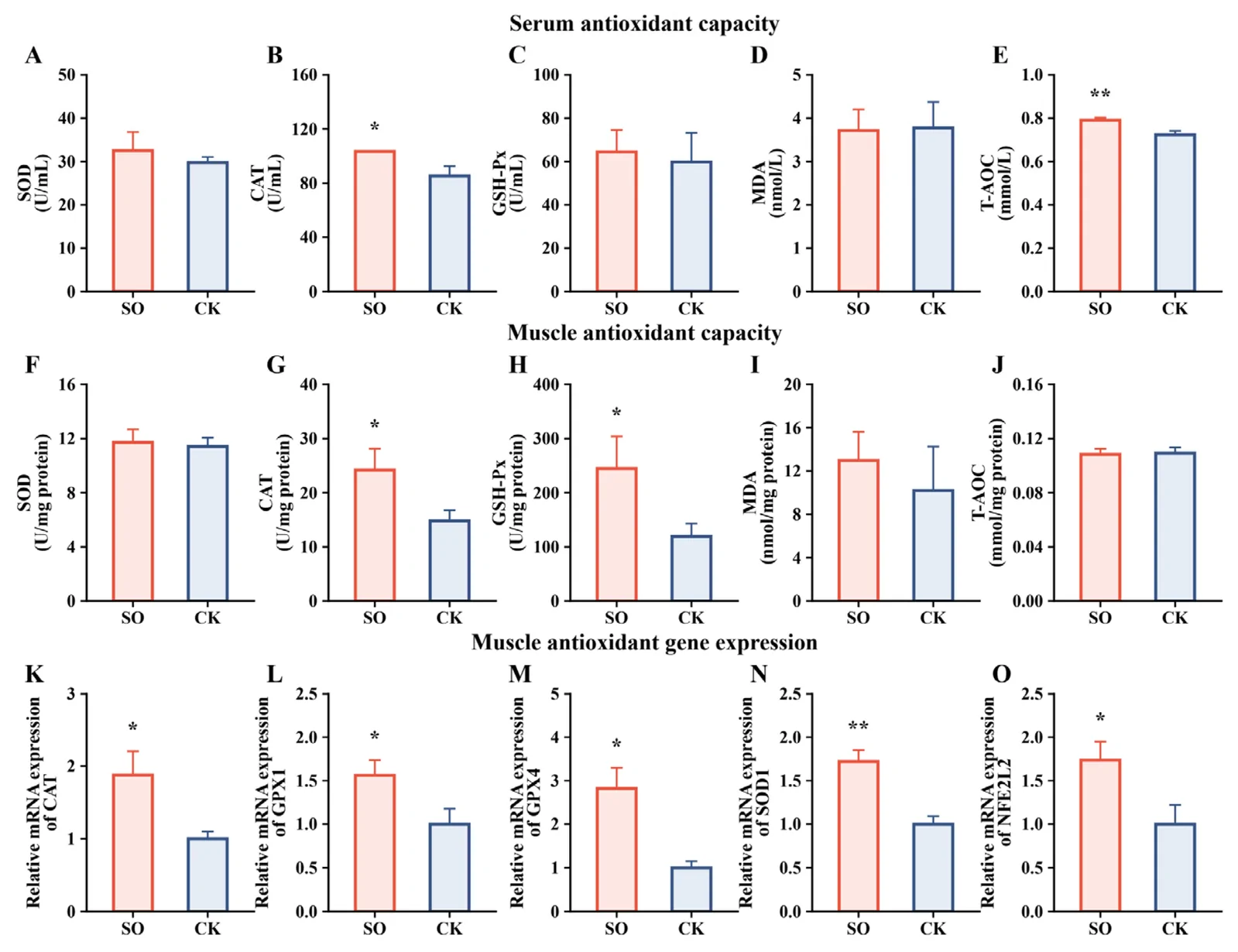
The antioxidant capacity in serum and *longissimus dorsi* muscles of *Hu* lambs fed CK or SO diets: (**A**–**E**) the serum levels of SOD, CAT, GSH-Px, MDA, and T-AOC; (**F**–**J**) the muscle levels of SOD, CAT, GSH-Px, MDA, and T-AOC; and (**K**–**O**) the relative mRNA expression of the antioxidant-related genes in the *longissimus dorsi* muscle, including *CAT*, *GPX1*, *GPX4*, *SOD1*, and *NFE2L2* Values are presented as the means ± SEM. * *p* < 0.05, ** *p* < 0.01. Abbreviations: MDA, malondialdehyde; T-AOC, total antioxidant capacity; CAT, catalase; SOD, superoxide dismutase; GSH-Px, glutathione peroxidase.

Figure 2 illustrates that the antioxidant capacity of *Hu* lambs differed between the CK and SO groups. In serum, CAT activity and T-AOC were significantly higher in the SO group than in the CK group (*p* < 0.05, Figure 2A–E). In muscle, CAT and GSH-Px activities were elevated in the SO group compared to the CK group (*p* < 0.05), whereas SOD activity, MDA level, and T-AOC showed no significant differences (*p* > 0.05, Figure 2F–J). At the level of the gene expression, the SO group showed the significantly upregulated mRNA expression of *CAT, GPX1, GPX4, SOD1,* and *NFE2L2* compared to the CK group (*p* < 0.05, Figure 2K–O).

### 3.5. The Detection of VOCs and the Selection of Key VOCs

**Figure 3:**
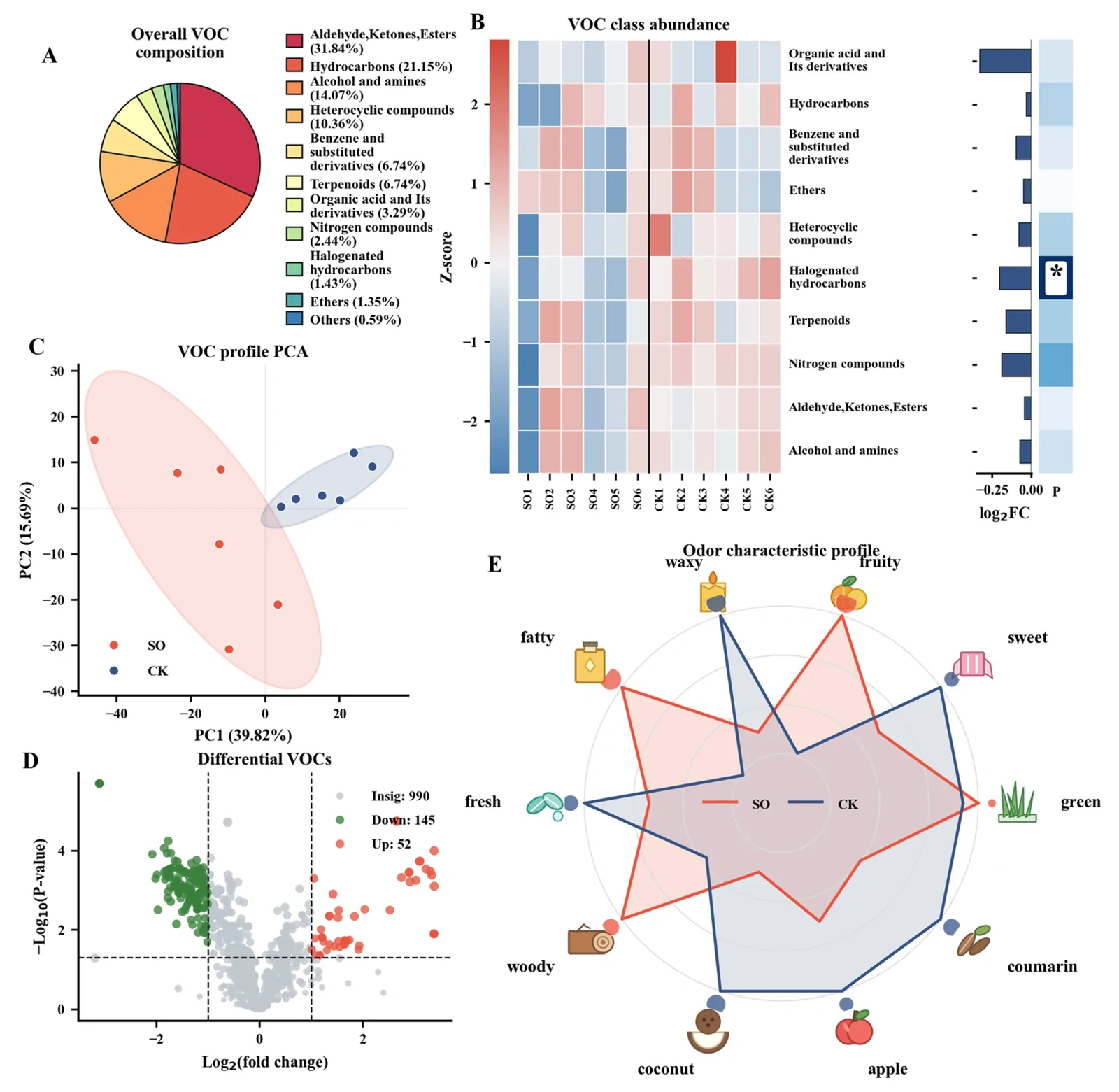
Volatile organic compound (VOC) profiles of *Hu* lambs fed SO or CK diets: (**A**) distribution of the identified VOCs; (**B**) heatmap obtained from cluster analysis; (**C**) PCA score plot of VOC profiles; (**D**) volcano plot of the differential VOCs; and (**E**) odor characteristic profiles of the SO and CK groups. * *p* < 0.05.

**Figure 4:**
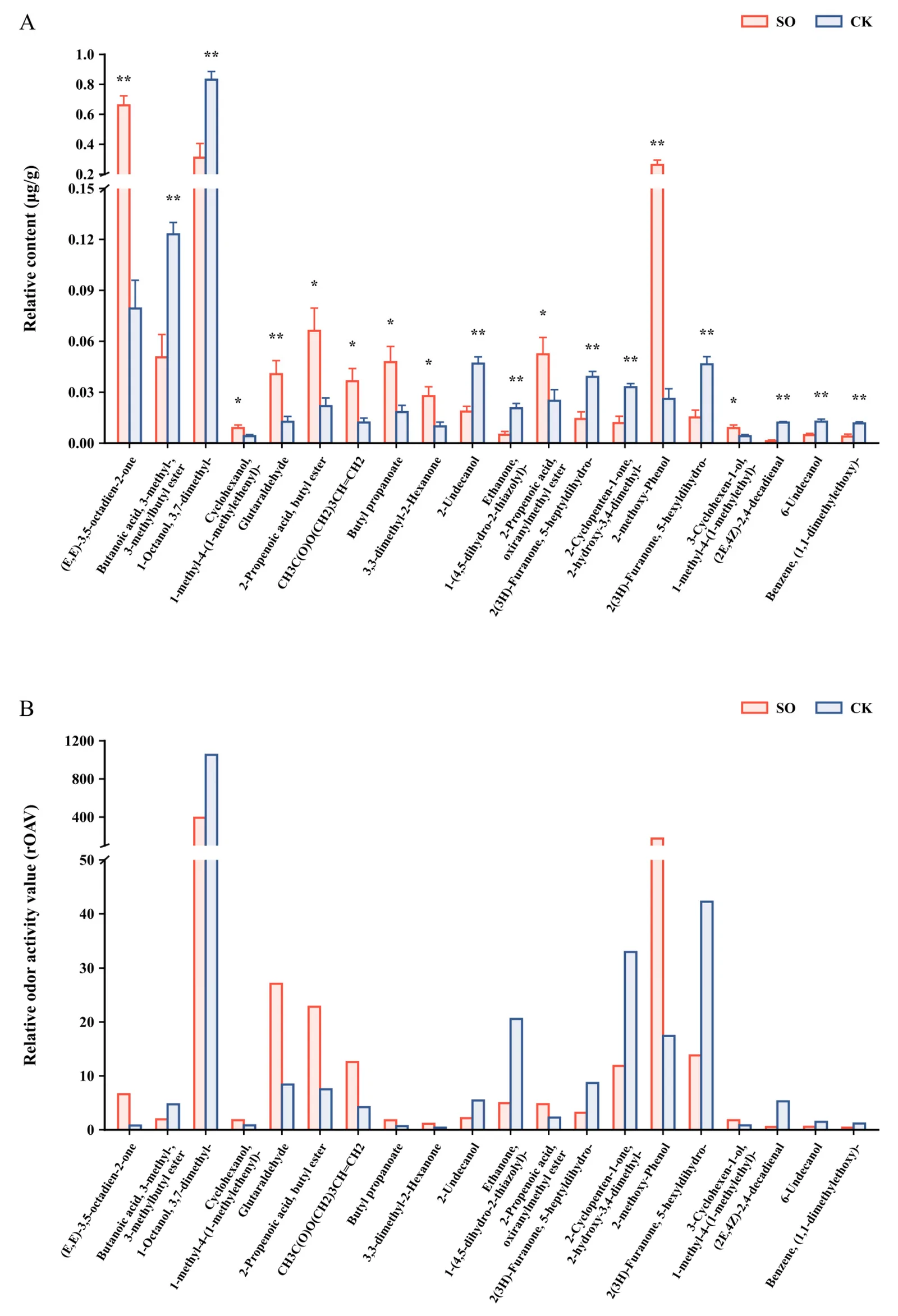
Relative contents and relative odor activity values (rOAVs) of key VOCs in the SO and CK groups: (**A**) relative content of the key VOCs; and (**B**) the rOAV. * *p* < 0.05, ** *p* < 0.01.

In total, 1187 VOCs were detected and categorized into 11 major classes (Figure 3A): aldehyde, ketones, esters, hydrocarbons, alcohol and amines, heterocyclic compounds, terpenoids, benzene and substituted derivatives, organic acids and their derivatives, nitrogen compounds, halogenated hydrocarbons, ethers, and others. Among these, aldehyde, ketones, and esters accounted for the majority of the VOCs (Figure 3A). A significant decrease in halogenated hydrocarbons was observed in the SO group (*p <* 0.05), while there were no significant differences (*p* > 0.05) in the other classes (Figure 3B). Although the VOCs of the two groups intersected through the principal component analysis (PCA), the differences between them were still obvious (Figure 3C). A total of 197 differential VOCs were identified, with 52 upregulated and 145 downregulated in the SO group compared with the CK group (Figure 3D). Correspondingly, green, fruity, and fatty flavor notes were enhanced in the SO group, whereas sweet and waxy notes were reduced (Figure 3E). Based on the multidimensional screening criteria, differential VOCs with rOAV > 1 were further defined as key VOCs. Figure 4 shows that 20 VOCs were recognized as key VOCs, among which 10 showed higher relative contents and 10 showed lower relative contents in the SO group than in the CK group (*p* < 0.05). Among these compounds, the relative contents of (E,E)-3,5-octadien-2-one and butyl propanoate were significantly higher in the SO group than in the CK group (*p* < 0.05, Figure 4A). The corresponding rOAVs of both compounds were also higher in the SO group than in the CK group (Figure 4B).

### 3.6. The Composition of FAs

Table S3 displayed the fatty acid profiles of *Hu* lambs on CK or SO diets. Although the total content of saturated fatty acids did not differ between the SO and CK groups (*p* > 0.05), the levels of C22:0 and C24:0 were significantly higher in the SO group (*p* < 0.01). For monounsaturated fatty acids and PUFAs, the SO group had significantly higher C18:1n9t and DHA contents (*p* < 0.01), whereas C15:1 content was significantly lower in the SO group (*p* < 0.05). Additionally, the total n-3 PUFA content and the n-3/n-6 PUFA ratio were significantly higher in the SO group (*p* < 0.01).

### 3.7. The Composition of Lipid

**Figure 5:**
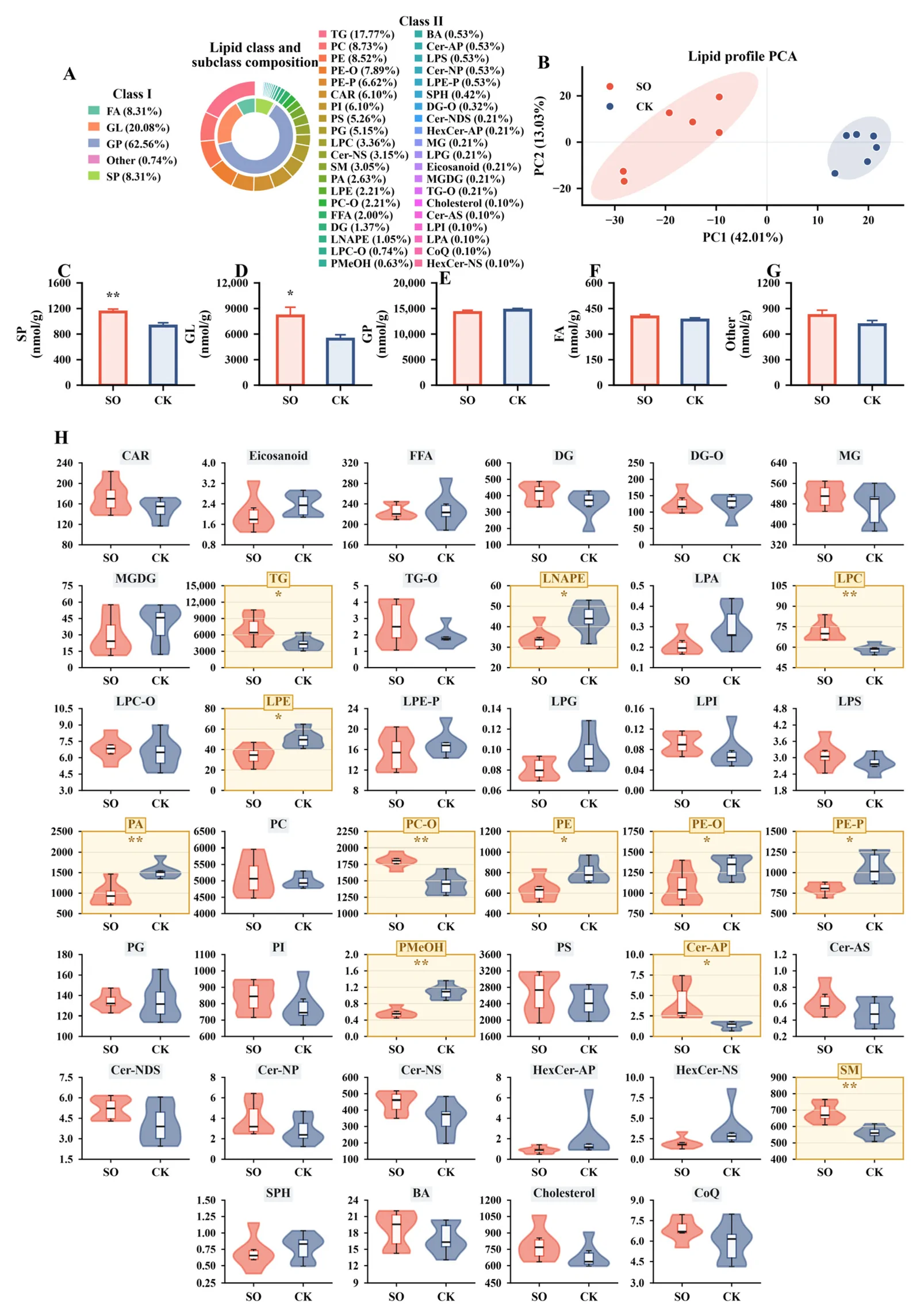
Lipid profiles of the *longissimus dorsi* muscle in *Hu* lambs fed SO or CK diets: (**A**) relative proportions of identified lipid classes; (**B**) PCA of the muscle lipidomic profiles, showing group separation; (**C**–**G**) comparison of the contents of major lipid class, including SP, GL, GP, FA, and other lipids; and (**H**) comparison of lipid subclass abundances between the two groups. In panel H, yellow background shading indicates lipid subclasses with significant differences between the SO and CK groups (*p* < 0.05). * *p* < 0.05, ** *p* < 0.01.

A total of 951 lipid metabolites in *longissimus dorsi* muscle were identified by lipidomics analysis. The identified class and subclass of lipids are shown in Figure 5A. In terms of the lipid class, glycerophospholipids (GP, 62.56%) were the predominant class, followed by glycerolipids (GL, 20.08%), sphingolipids (SP, 8.31%), fatty acyls (FA, 8.31%), and other class (0.74%). These lipids were categorized into 40 subclasses, where triacylglycerols (TG, 17.77%) showed the highest abundance (Figure 5A). PCA showed that samples from the SO and CK groups were separated based on their lipid profiles (Figure 5B). GL and SP levels were significantly higher in the SO group than in the CK group (*p* < 0.05, Figure 5C–G). Among 40 lipid subclasses, the level of TG, Cer-AP, LPC, PC-O, and SM increased significantly in the SO group, while LNAPE, LPE, PA, PE, PE-O, PE-P, and PMeOH decreased markedly (*p* < 0.05, Figure 5H).

### 3.8. Identification of DLMs

**Figure 6:**
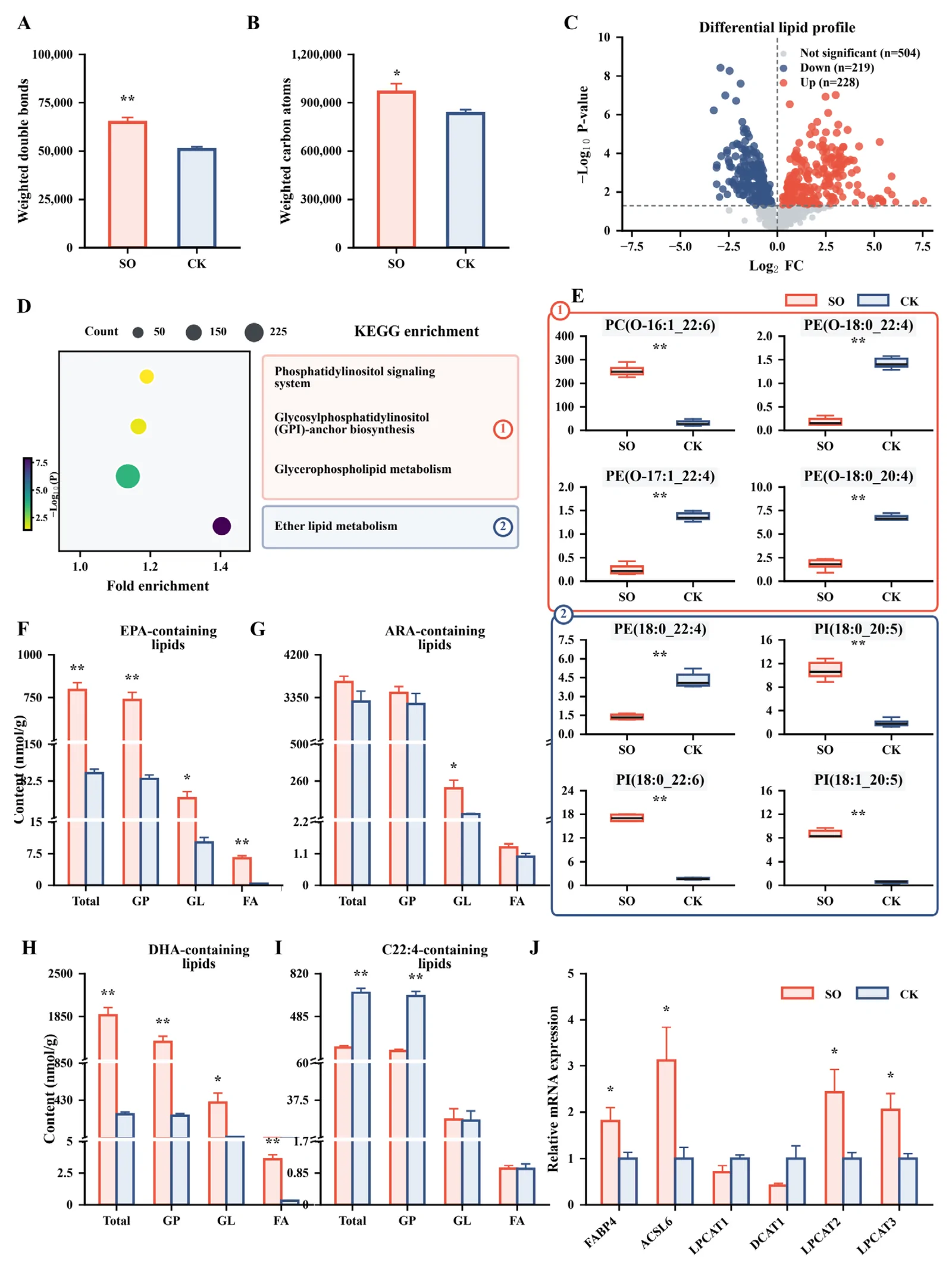
Identification of differential lipid metabolites (DLMs) between the SO and CK groups: (**A**) total number of double bonds in fatty acids; (**B**) total number of carbon atoms in fatty acids; (**C**) volcano plot of DLMs between the two groups; (**D**) KEGG enrichment analysis of lipid metabolism-related pathways; (**E**) the top four DLMs with the lowest *p* values in each of the two pathway groups: ① glycerophospholipid metabolism-related pathways and ② ether lipid metabolism pathway; (**F**–**I**) contents of EPA, ARA, DHA, and C22:4 containing lipids, respectively; and (**J**) expression levels of lipid-metabolism-related genes in the SO and CK groups. * *p* < 0.05, ** *p* < 0.01.

The total numbers of double bonds and carbon atoms in lipid molecules were significantly higher in the SO group than in the CK group (*p* < 0.05, Figure 6A,B). Among the 951 lipid molecules, a total of 447 lipid species were identified as DLMs, including 228 upregulated and 219 downregulated in the SO group (Figure 6C). KEGG pathway enrichment analysis revealed four significantly enriched lipid metabolism pathways. They were categorized into two major groups: (1) glycerophospholipid-metabolism-related pathways, including glycerophospholipid metabolism, glycosylphosphatidylinositol (GPI)-anchor biosynthesis, and the phosphatidylinositol signaling system, and (2) the ether lipid metabolism pathway (Figure 6D). The top four DLMs with the lowest *p*-values in the major pathway group were selected for further analysis. In the SO group, significantly higher levels of n-3 PUFA-enriched lipids, including PC(O-16:1/22:6), PI(18:0/22:6), PI(18:1/20:5), and PI(18:0/20:5), and lower levels of n-6 PUFA-enriched lipids, including PE(O-18:0/22:4), PE(O-17:1/22:4), PE(O-18:0/20:4), and PE(18:0/22:4), were observed (Figure 6E). A further analysis showed higher DHA and EPA levels in total lipids and all three lipid classes (GP, GL, and FA), and lower C22:4 in total lipids and GP in the SO group (*p* < 0.05, Figure 6F–I). The SO diet increased the relative mRNA abundance of *FABP4*, *ACSL6*, *LPCAT2,* and *LPCAT3* relative to the CK diet (*p* < 0.05, Figure 6J).

### 3.9. Identification of Candidate Lipid Biomarkers Based on Machine Learning and Spearman Correlation

**Figure 7:**
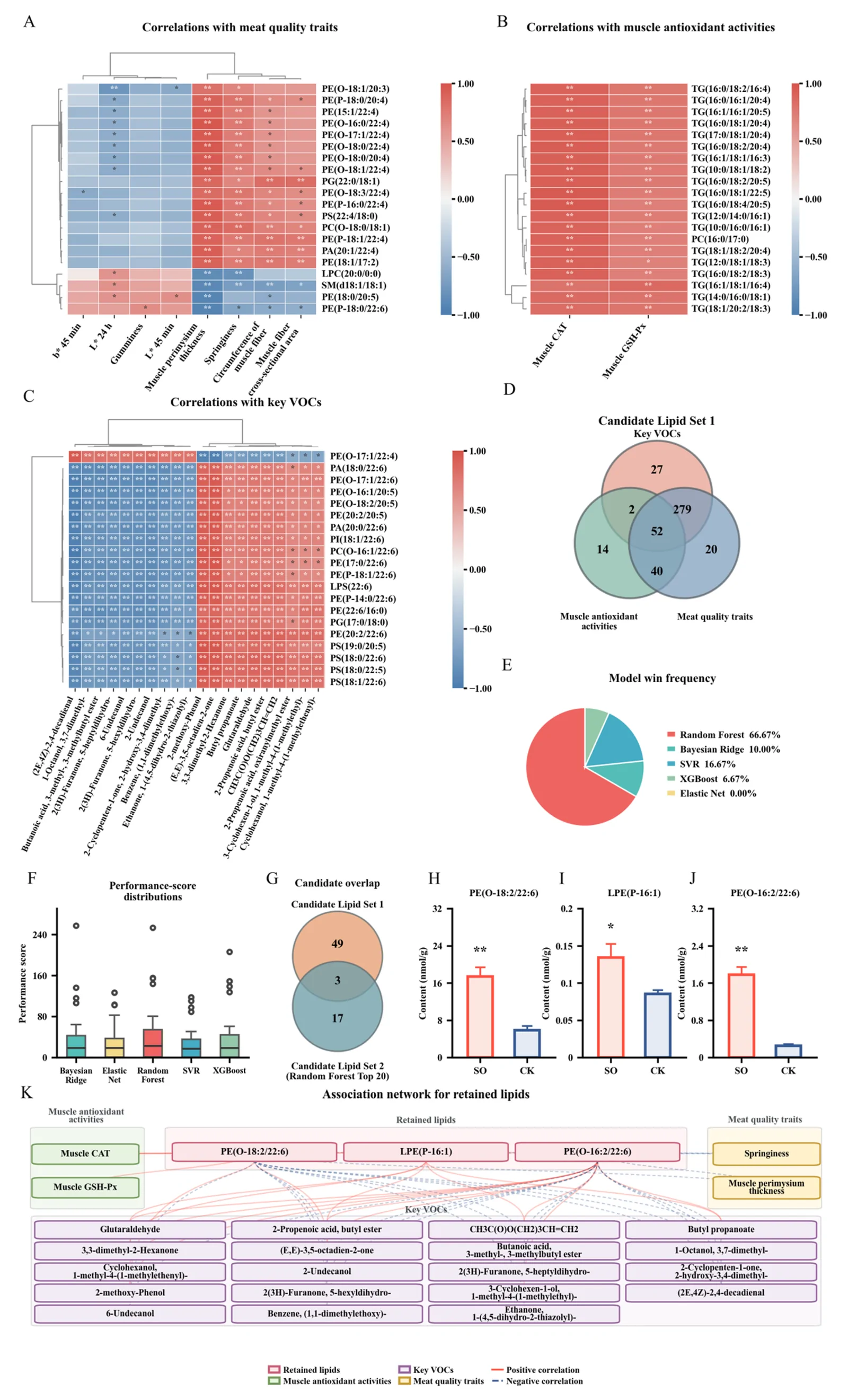
Screening and identification of candidate lipid biomarkers using Spearman correlation and machine learning: (**A**–**C**) Spearman correlations between DLMs and differential meat quality traits, differential antioxidant enzyme activities, and key VOCs, respectively; (**D**) Venn diagram showing the overlap among the three DLM sets identified by Spearman correlation analysis; (**E**) percentage of variables for which each regression model ranked first; (**F**) distribution of the performance scores of the five regression models across the 30 evaluated variables; (**G**) Venn diagram showing the overlap between Candidate Lipid Set 1, identified by Spearman correlation analysis, and Candidate Lipid Set 2, selected using the Random Forest model; (**H**–**J**) contents of PE(O-18:2/22:6), LPE(P-16:1), and PE(O-16:2/22:6), respectively; and (**K**) Spearman correlation network between the three CLBs and differential meat quality traits, differential antioxidant enzyme activities, and key VOCs. * *p* < 0.05, ** *p* < 0.01.

Spearman correlation analysis identified 391, 108, and 360 DLMs strongly correlated with differential meat quality traits, differential antioxidant enzyme activities, and key VOCs, respectively (|r| > 0.7, *p* < 0.05). Several C22:4-containing DLMs were negatively correlated with L* and positively correlated with springiness and muscle fiber cross-sectional area (Figure 7A). DLMs strongly correlated with CAT and GSH-Px activities were mainly TG species (Figure 7B). Several DHA-containing DLMs were positively correlated with key VOCs, including (E,E)-3,5-octadien-2-one and butyl propanoate, whereas several C22:4-containing DLMs were correlated with 2-undecanol (Figure 7C). A total of 52 DLMs were shared among the three sets and were designated as Candidate Lipid Set 1 (Figure 7D).

Secondly, five regression models (Bayesian Ridge Regression, Elastic Net Regression, Random Forest Regression, SVR, and XGBoost Regression) were then used to evaluate the relationships between DLMs and the differential meat quality traits, differential antioxidant enzyme activities, and key VOCs. As shown in Figure 7E, Random Forest ranked first for 20 of the 30 evaluated variables (66.67%), showing the highest win frequency among the five regression models (Figure 7E). The overall performance of the five models is shown in Figure 7F, and Random Forest also had the lowest mean rank across the 30 variables (1.37, Supplementary Figure S1). Based on these results, Random Forest was selected for further analysis. Its performance across 100 repeated train/test splits was further evaluated using MAE and RMSE, and the corresponding means, standard deviations, and 2.5th and 97.5th percentiles are provided in Supplementary Table S4. DLMs were ranked according to their feature importance scores, and the top 20 were designated as Candidate Lipid Set 2.

Thirdly, the intersection between Candidate Lipid Sets 1 and 2 contained three DLMs (Figure 7G), which were designated as CLBs. These lipids were PE(O-18:2/22:6), LPE(P-16:1), and PE(O-16:2/22:6), and their contents are shown in Figure 7H and Figure 7I, and 7J, respectively. All three CLBs showed significantly higher contents in the SO group than in the CK group (*p* < 0.05). The correlations of these CLBs with differential meat quality traits, differential antioxidant enzyme activities, and key VOCs are shown in Figure 7K.

## 4. Discussion

In this study, the initial and final body weights were similar between the SO and CK groups. However, the SO group had a higher ADG during the late fattening period (days 57–84), with an increasing trend across the whole trial. This agrees with previous sheep studies, suggesting that SO supplementation has no negative effect on growth performance [11]. The improvement in late-stage ADG may be associated with rumen microbial changes and enhanced nutrient conversion efficiency [23].

To clarify the mechanistic basis underlying SO-induced improvements in meat quality, we first examined whether SO drives the coordinated remodeling of muscle lipid metabolism. In this study, the overall unsaturation and carbon chain length of lipid molecules significantly increased in the SO group. This finding is consistent with the noted increases in DHA and EPA, along with enhancements in total n-3 PUFA concentrations and the n-3/n-6 ratio in the overall fatty acid composition. In line with previous studies, these results confirm the successful deposition of exogenous n-3 PUFAs and their active incorporation into lipid structures [12]. At the lipid class level, the GL abundance was greater in the SO group, with TG also showing a marked increase. This reflects an enhanced capacity for energy storage and intramuscular lipid deposition in muscle tissue [5]. Meanwhile, elevated SP levels suggest potential changes in the membrane lipid structural complexity and associated functions [24]. Collectively, these findings indicate that SO-induced lipid remodeling is not merely a simple lipid accumulation but may also involve changes in the distribution of fatty acids among different lipid classes. The significant upregulation of *FABP4*, *ACSL6, LPCAT2*, and *LPCAT3* in the SO group may be associated with the selective esterification and integration of n-3 PUFAs into membrane lipids. Specifically, a high FABP4 expression promotes the preferential intracellular transport of DHA [25]. ACSL6 efficiently activates DHA into DHA-CoA, providing the substrate for downstream reactions [26]. Subsequently, *LPCAT2* and *LPCAT3* use DHA-CoA as an acyl donor to incorporate DHA into lysophospholipids at sn-2 [27], thereby driving the synthesis of n-3 PUFA-containing phospholipids. Various phospholipid species containing DHA or EPA significantly increased within the PC and PI classes in the SO group. Conversely, species rich in n-6 PUFAs experienced a notable decrease within the PE class. For example, the upregulation of PC(O-16:1/22:6) and the downregulation of PE(O-18:0/22:4) exemplify a typical competitive displacement in membrane phospholipids [28]. This pattern corresponds with the increase in DHA and the decrease in C22:4 observed in overall the fatty acid fraction, suggesting that these gene expression changes may be related to the observed lipid remodeling. These changes in lipid composition may provide a biochemical and structural basis for the observed changes in meat quality, oxidative stability, and flavor.

Dietary supplementation with SO exerts a cascading influence on meat quality traits by altering the lipid composition of muscle cell membranes. The SO group exhibited an increased overall unsaturation and carbon chain length in lipid molecules, alongside a specific remodeling of membrane-associated lipids. Within sphingolipid subclasses, Cer-AP and SM were significantly upregulated. Simultaneously, DHA-containing phospholipid species were enriched in PC and PE, with a notable increase in PC(O-16:1_22:6). Previous research has shown that DHA incorporation into phospholipids alters the membrane fluidity, permeability, and protein function, thereby modifying the hydration and functional state of the cell membrane [29]. Specifically, Cer-AP demonstrates a strong hydrogen-bonding potential, which may influence lipid packing and membrane organization [30]. Meanwhile, changes in the SM content may influence the mechanical properties of muscle tissue [24]. In the SO group, increased levels of LPC were accompanied by reduced springiness and increased gumminess, while elevated L* values were observed in meat samples at both 45 min and 24 h postmortem. These changes may improve the appearance of raw meat and alter its textural properties [31]. According to Khliji et al. [32], instrumental lightness (L*) is associated with consumer evaluation and acceptance of the fresh lamb color. Therefore, the increased L* values observed in the SO group may indicate a more favorable visual appearance of the meat.

Furthermore, membrane lipid remodeling coincided with alterations in the muscle fiber structure and type. SO feeding increased the mRNA abundance of *MyHCI* and *MYOG* but reduced that of *MyHCIIb* and *MyHCIIx*. This expression pattern suggests a transition from fast-twitch to slow-twitch fibers [33]. Smaller muscle fibers may offer less physical resistance, thereby contributing to a lower tissue elasticity and potentially improving tenderness. These findings align with the research conducted by Maddock et al. [34], which indicated that feeding cattle a diet high in n-3 PUFA significantly enhances the meat tenderness.

n-3 PUFAs, especially DHA, are highly prone to oxidation because of their multiple bis-allylic hydrogens. However, MDA levels did not increase after SO supplementation in either serum or muscle. Meanwhile, DHA-containing phospholipids were enriched in the SO group, showing that the increase in DHA was accompanied by its incorporation into phospholipids in an esterified form. When DHA enters membrane phospholipids, it changes the membrane’s physical properties and microenvironment [35]. This shift can limit the direct contact with external pro-oxidants, helping to maintain oxidative stability. Previous studies show that DHA acts as an antioxidant in animal models and cells, primarily by regulating antioxidant systems at both the signaling and transcriptional levels [36]. In serum, CAT activity and T-AOC were significantly higher in the SO group. In muscle, CAT and GSH-Px activities increased significantly in the SO group. This suggests that the higher CAT and GSH-Px activities may enhance hydrogen peroxide removal in muscle. The gene expression partly agreed with these biochemical results. The SO group showed higher mRNA levels for *CAT*, *GPX1*, *GPX4*, and *SOD1*, alongside an increased *NFE2L2* expression. These changes suggest that NFE2L2-related regulation may be involved in the antioxidant response [36]. Importantly, the intake of natural antioxidants from the raw SO material may have additionally contributed to this oxidative stability [37].

Lipids act as flavor precursors. During heating and oxidation, they degrade into volatile compounds such as aldehydes, ketones, alcohols, and esters. The CK group had higher levels of (2E, 4Z)-2, 4-decadienal, a typical oxidation product of linoleic and C22:4, often associated with paint-like or oxidized fat off-flavors [38]. The SO group had a lower proportion of n-6 PUFA and may have reduced the substrates available for lipid oxidation and, thereby, limited the formation of undesirable aldehydes [39]. This may have helped reduce the off-flavor formation. Furthermore, the level of 2-undecanol decreased in the SO group. This spoilage-related alcohol is typically linked to off-flavors in meat [40], so its reduction may also have contributed to the changes in the flavor profile. Conversely, the SO group showed increased levels of TGs and related subclasses in its lipid pool, which may be associated with the formation of lipid-derived aroma compounds [41]. (E,E)-3,5-Octadien-2-one, a volatile product derived from n-3 PUFA oxidation, was significantly increased in the SO group. This compound has been associated with grassy and fruity notes [41]. Similarly, butyl propanoate, a compound associated with fruity and apple-like notes, showed a higher relative content in the SO group. This finding is broadly consistent with previous reports that dietary DHA supplementation can alter odor profiles and improve taste-related attributes [42]. Therefore, SO feeding reduced the substrate supply for n-6-related off-flavor aldehydes while increasing the availability of n-3-related substrates and lipid reserves. This shifted the lamb’s flavor profile toward greener, fattier, and fruitier characteristics.

Spearman correlation and Random Forest analyses were combined to screen lipid species associated with changes in the meat quality. A total of three CLBs were identified and found to be significantly upregulated in the SO group: PE(O-18:2/22:6), LPE(P-16:1), and PE(O-16:2/22:6). The three identified candidate biomarkers are phospholipids. These structural lipids are important for maintaining membrane fluidity and lipid–protein interactions [43]. Therefore, the increased abundance of DHA-containing PE molecules may be associated with changes in the meat texture. Furthermore, plasmalogens contain vinyl ether bonds that have been suggested to contribute to the antioxidant protection of membrane components [44]. Collectively, these three lipid species may represent candidate indicators of SO-induced lipid remodeling and its associations with meat quality, antioxidant capacity, and flavor. However, the additional functional validation of these three identified lipid species and validation of the associated regulatory pathways at the protein expression level are needed to further substantiate their biological significance.

Nevertheless, the relatively small sample size of the present study may limit the statistical power of the correlation analysis and increase the risk of overfitting in machine-learning models. The variation in prediction errors across repeated train/test splits further indicates that the model performance was sensitive to data partitioning in the present limited cohort. Therefore, the CLBs identified in this study remain exploratory. Future studies involving larger sample sizes and independent validation cohorts, together with targeted lipidomic quantification or functional validation, are required to confirm their biological relevance and improve model robustness, predictive accuracy, and generalizability.

## 5. Conclusions

The present study indicates that the dietary supplementation with *Schizochytrium* oil (SO) may alter the meat quality, oxidative stability, and flavor profile of *Hu* lambs by driving a targeted remodeling of muscle lipid metabolism. Specifically, SO promotes the deposition of n-3 PUFAs (DHA and EPA) while reducing n-6 PUFA-enriched lipids. These lipids were associated with an improved lightness, antioxidant defenses, and desirable aroma attributes. Furthermore, integrated Spearman correlation and random forest modeling identified PE(O-18:2/22:6), LPE(P-16:1), and PE(O-16:2/22:6) as candidate lipid biomarkers. Overall, SO supplementation represents a promising nutritional strategy to rebalance the muscle fatty acid composition and improve the selected quality attributes of lamb meat in intensive production systems.

## Figures and Tables

**Table 1:** The effects of dietary CK or SO diets on growth performance of *Hu* lambs.

Item	CK	SO	SEM	*p*-Value
Initial BW (kg)	21.93	21.75	0.98	0.86
BW on day 28 of feeding (kg)	29.76	29.73	1.39	0.99
BW on day 56 of feeding (kg)	40.77	40.99	1.87	0.91
Final BW (kg)	46.48	49.27	2.12	0.23
Overall ADG (kg/day)	0.29	0.33	0.02	0.06
ADG on days 0–28 (kg/day)	0.28	0.28	0.02	0.82
ADG on days 29–56 (kg/day)	0.39	0.40	0.03	0.74
ADG on days 57–84 (kg/day)	0.20	0.30	0.02	<0.01

Note: BW, body weight; ADG, average daily gain.

**Table 2:** The effects of dietary CK or SO diets on meat quality traits of *Hu* lambs.

Item	CK	SO	SEM	*p*-Value
pH 45 min	6.44	6.39	0.09	0.60
pH 24 h	5.43	5.46	0.03	0.44
Drip loss %	4.17	4.16	0.66	0.99
Press loss %	35.46	37.86	0.97	0.10
Cooking loss %	40.52	43.36	2.14	0.21
L* 45 min	32.67	35.68	1.12	0.02
a* 45 min	15.08	15.44	0.59	0.55
b* 45 min	4.61	5.35	0.34	0.05
L* 24 h	39.34	41.29	0.86	0.04
a* 24 h	19.08	19.16	0.67	0.91
b* 24 h	12.39	12.52	0.60	0.83
Shear force N	74.74	73.55	7.35	0.87
Hardness	29.48	28.59	5.85	0.88
Adhesiveness	0.05	0.06	0.01	0.42
Cohesiveness	0.46	0.48	0.03	0.47
Springiness	2.47	2.11	0.13	0.02
Gumminess	1.86	2.90	0.26	0.01
Chewiness	4.70	6.64	1.11	0.13

Note: CK, control group; SO, *Schizochytrium* oil group; L*, lightness; a*, redness; b*, yellowness; SEM, standard error of the mean.

## Data Availability

The original contributions presented in this study are included in the article/Supplementary Materials. Further inquiries can be directed to the corresponding author.

## Supplementary Materials

The following supporting information can be downloaded at: https://www.mdpi.com/article/10.3390/foods15203753/s1, Figure S1: Distribution of regression model ranks across the 30 evaluated phenotypes. For each phenotype, the five regression models were ranked according to their performance score, with rank 1 representing the best-performing model and rank 5 representing the lowest-performing model. Each point represents the rank of one model for one phenotype. Mean ranks are shown above each model. Random Forest showed the lowest mean rank (1.37) among the five models; Table S1: Ingredient composition, analyzed nutrient levels, and fatty acid composition of the CK and SO diets; Table S2: Primer sequences for quantitative qRT-PCR; Table S3: Fatty acid composition of *longissimus dorsi* muscle from *Hu* lambs fed CK or SO diets; Table S4: Prediction performance of the five regression models for the 30 evaluated phenotypes across 100 repeated train/test splits.
