# Supplementary material for: Integrative Lipidomics Reveals Schizochytrium Oil-Induced Muscle Lipid Remodeling Associated with Meat Quality, Oxidative Stability, and Flavor in Hu Lambs
Source: Foods. 2026 Oct 21;15(20):3753. doi: 10.3390/foods15203753 (PMC13704049; doi:10.3390/foods15203753)
Supplement: Supplementary file 1 [file foods-15-03753-s001.zip › foods-4530271-supplementary.pdf]

Supplementary Material

Integrative Lipidomics Reveals Schizochytrium-Oil-Induced Muscle Lipid Remodeling Associated with Meat Quality, Oxidative Stability, and Flavor in *Hu* Lambs

Rongyu Yao [1], Haiyu Ma [1,2], Danian Chen [3], Xiaoli Zhou [3], Peidi Zhao [1], Xiuxiu Weng [1] and Wanhong Li [1,*]

[1] State Key Laboratory of Herbage Improvement and Grassland Agro-Ecosystems, Key Laboratory of Grassland Livestock Industry Innovation, Ministry of Agriculture and Rural Affairs, Engineering Research Center of Grassland Industry, Ministry of Education, College of Pastoral Agriculture Science and Technology, Lanzhou University, Lanzhou 730020, PR China

[2] College of Veterinary and Animal Science, Dingxi Vocational and Technical College, Dingxi 730500, PR China

[3] Dongxiang County Mutton Sheep Industry Research Center, Dongxiang, 731400, PR China;

***Corresponding Author: Wanhong Li**

Tel: +86-0931-891-4266, Fax: +86-0931-891-4266, E-mail: limh@lzu.edu.cn

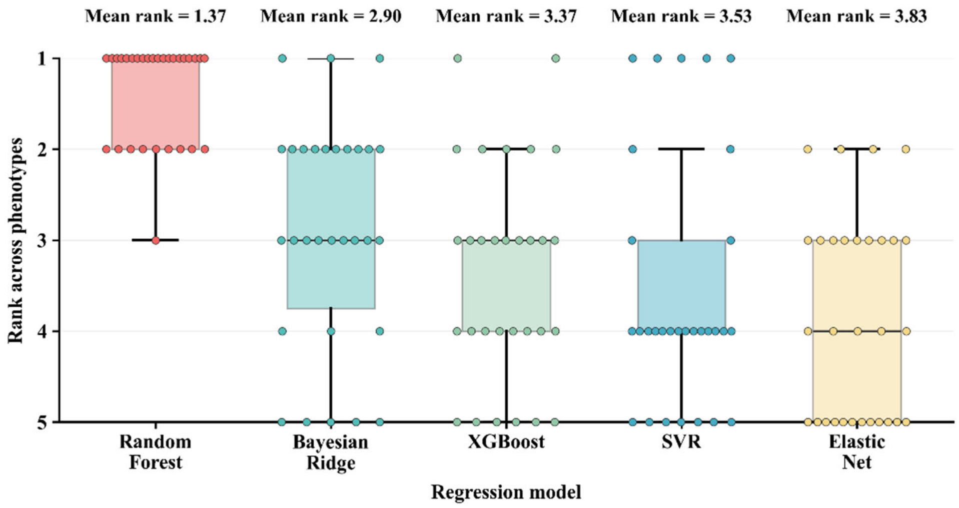

Supplementary Figure S1. Distribution of regression model ranks across the 30 evaluated phenotypes. For each phenotype, the five regression models were ranked according to their performance score, with rank 1 representing the best-performing model and rank 5 representing the lowest-performing model. Each point represents the rank of one model for one phenotype. Mean ranks are shown above each model. Random Forest showed the lowest mean rank (1.37) among the five models.

Supplementary Table S1. Ingredient composition, analyzed nutrient levels, and fatty acid composition of the CK and SO diets.

Items	CK	SO
**Ingredients** (%)		
Corn stover	24	24
Corn husks	8	8
Cracked corn	26	26
Soybean meal	5	5
Cottonseed meal	6	6
Corn germ meal	20	20
Palm oil	4	3
Schizochytrium oil	0	1
Puffed urea	1	1
Molasses	3	3
NaCl	0.5	0.5
Limestone	1.2	1.2
Sodium bicarbonate	0.8	0.8
Vitamin premix	0.5	0.5
Total	100	100
**Nutrients** (%)		
DM	92.26	92.11
Ash content	6.94	6.91
CF	5.58	5.45
NDF	31.43	31.76
ADF	15.60	14.88
CP	15.42	15.31
**Fatty acids** (%)		
C12:0	0.30	0.26
C14:0	0.73	0.75
C16:0	50.67	48.32
C16:1	0.15	0.15
C17:0	0.12	0.13
C18:0	8.27	6.97
C18:1n9t	8.95	7.93

C18:1n9c	0.00	0.78
C18:2n6t	24.96	24.68
C20:0	0.29	0.31
C18:3n6	0.04	0.06
C18:3n3	2.63	2.48
C20:2	0.07	0.09
C22:0	0.21	0.21
C20:3n6	0.04	0.07
C20:3n3	0.12	0.17
C22:2	0.13	0.21
C24:0	0.12	0.14
C20:5n3	0.03	0.09
C22:6n3	2.18	6.20
ΣSFA	60.71	57.09
ΣMUFA	9.10	8.86
ΣPUFA	30.19	34.04
Σn-3 PUFA	4.95	8.93
Σn-6 PUFA	25.04	24.81
Σn-3/n-6 PUFA	0.20	0.36

Abbreviations: DM, dry matter; NDF, neutral detergent fibers; ADF, acidic detergent fibers; CP, crude protein, CF: crude fat.

Supplementary Table S2. Primer sequences for quantitative qRT-PCR.

Primer	Primer sequences (5′→3′)	Sizes (bp)
ACSL6	F: GCATTTTCGGGGCTGTATGC	111
XM_042250258.2	R: GTTCATGGCGTCCAAAATCTCT	
CAT	F: CCGCGCAGAAACCTGATGT	196
XM_060400054.1	R: AAGTAGCCAAAAGCCCCTGC	
DGAT1	F: CGTGCGTGATGAGAAGGTCAA	90
XM_060419895.1	R: GGGTCATCCAGGTACCCTTTG	
FABP4	F: ACTTGAAAGTGAGGTGGCCC	84
NM_001114667.1	R: GTTTTCCCGTCACACTTGCC	
GAPDH	F: TCGGAGTGAACGGATTTGGC	178
NM_001190390.1	R: CCGTTCTCTGCCTTGACTGT	
GPX1	F: CAGTTTGGGCATCAGGAAAAC	125
XM_004018462.5	R: TTCTCGCCATTCACCTCGC	
GPX4	F: CATGCTGGGAAACGCCATCA	159
XM_060416444.1	R: GAAGAGGGTGAGGGACGACT	
LPCAT1	F: GAGTACGTGGTGGCCCTGTC	87
XM_027980187.3	R: CTGTGACCCGAACATCTTGAAC	
LPCAT2	F: GCATTGTGCACCAGGTCTTTCC	172
XM_042231639.2	R: CCGAGGCTCTGCTTTCACTTT	
LPCAT3	F: AACTCACGGTTCTGCACTCC	127
XM_027967922.3	R: GTAGACCAGAGGGACACCAAC	
MyHCI	F: GAACAGGCCAACACCAACCT	145
XM_004010325.4	R: CCTCATTCAAGCCCTTGGCG	
MyHCIIa	F: GAGGAACAATCCAATACAAATCTATCT	173
XM_027974884.3	R: CCCATAGCATCAGGACACGA	
MyHCIIb	F: GGAGGAACAATCCAATGTCAAC	178
XM_060395436.1	R: GTCACTTTTTAGCATTTGGATGAGTTA	
MyHCIIx	F: GCAGCGCCATCCAGTACATA	178
XM_004012706.5	R: CATTCACTGGGCACCCCTG	
MYOG	F: GGCACCTCTGGGAGTTTGTC	118
NM_001174109.1	R: CAGGAATGTCCCACTGGAGTC	
NFE2L2	F: TCCGTTATCTCATGGGGGAGA	89
XM_015093345.4	R: CTCCACTCCATACAGGCACACTA	

SOD1<br>NM_001145185.2	F: GCGTTTCCTTGTCTGCTTGG<br>R: ACGACTTTATCTCCCTTTGCCT	153

Abbreviations: *ACSL6*: acyl-CoA synthetase long chain family member 6, *CAT*: catalase, DGAT1: diacylglycerol O-acyltransferase 1, *FABP4*: fatty acid binding protein 4, *GAPDH*: glyceraldehyde-3-phosphate dehydrogenase, *GPX1*: glutathione peroxidase 1, *GPX4*: glutathione peroxidase 4, *LPCAT1*: lysophosphatidylcholine acyltransferase 1, *LPCAT2*: lysophosphatidylcholine acyltransferase 2, *LPCAT3*: lysophosphatidylcholine acyltransferase 3, *MyHCI*: myosin-7, *MyHCIIa*: myosin heavy chain 2, *MyHCIIb*: myosin-4, *MyHCIIx*: myosin heavy chain 1, *MYOG*: myogenin, *NFE2L2*: NFE2 like bZIP transcription factor 2, *SOD1*: superoxide dismutase 1.

Supplementary Table S3. Fatty acid composition of *longissimus dorsi* muscle from *Hu* lambs fed CK or SO diets.

Fatty acids (mg/100g)	CK	SO	SEM	*P*-Value
C4:0	2.89	2.78	0.57	0.84
C6:0	9.84	7.33	5.21	0.64
C8:0	0.47	0.92	0.19	0.04
C10:0	6.04	10.23	2.13	0.08
C12:0	4.70	5.61	1.00	0.38
C13:0	0.19	0.19	0.04	0.96
C14:0	104.88	119.56	24.24	0.55
C15:0	5.08	6.16	1.20	0.38
C16:0	959.32	1254.36	179.65	0.12
C18:0	606.15	580.46	109.06	0.82
C20:0	3.72	3.89	0.81	0.83
C22:0	1.03	10.16	0.75	< 0.01
C24:0	4.51	37.51	2.14	< 0.01
ΣSFA	1708.83	2039.15	305.33	0.30
C14:1	1.87	1.66	0.31	0.51
C15:1	7.59	4.72	0.63	< 0.01
C16:1	55.93	54.72	8.07	0.88
C17:1	10.19	10.62	1.70	0.81
C18:1n9t	90.73	185.36	21.34	< 0.01
C18:1n9c	1336.93	1460.12	210.04	0.57
C20:1n9t	12.92	5.62	8.27	0.40
C22:1n9c	7.18	10.93	8.39	0.66
C24:1n9	0.94	1.55	0.68	0.39
ΣMUFA	1524.26	1735.29	238.01	0.39
C20:2	9.28	13.12	10.94	0.73
C22:2n6t	4.87	7.59	5.99	0.66
C18:2n6c	172.79	148.44	13.95	0.10
C18:3n3	8.74	1.16	6.38	0.27
C20:3n6	5.44	3.80	0.85	0.07
C20:3n3	48.15	39.28	4.06	0.05
C18:3n6	1.30	1.20	0.16	0.53
C20:4n6	0.79	1.74	0.32	0.01
C20:5n3	0.28	0.53	0.28	0.39
C22:6n3	6.70	47.28	3.00	< 0.01
PUFA	258.36	264.15	31.34	0.86
Σn-3 PUFA	63.88	88.26	6.33	< 0.01
Σn-6 PUFA	185.20	162.77	18.78	0.25
Σn-3/Σn-6 PUFA	0.35	0.55	0.03	< 0.01

Abbreviations: FA, fatty acid; SFA, saturated fatty acid; MUFA, monounsaturated fatty acid; PUFA, polyunsaturated fatty acid.

Supplementary Table S4. Prediction performance of the five regression models for the 30 evaluated phenotypes across 100 repeated train/test splits.

Phenotype	Model	MAE, mean ± SD	MAE empirical interval	RMSE, mean ± SD	RMSE empirical interval	Performance score (rank)
(2E,4Z)-2,4-decadienal	Bayesian Ridge	0.00177 ± 0.000897	0.000595–0.00353	0.00212 ± 0.0011	0.00067–0.00432	257 (1)
	Random Forest	0.0019 ± 0.000621	0.000894–0.00337	0.00205 ± 0.000597	0.00106–0.0034	253 (2)
	XGBoost	0.0022 ± 0.00185	0.000414–0.00782	0.00266 ± 0.00225	0.000506–0.00873	206 (3)
	SVR	0.00552 ± 0.000782	0.00409–0.00673	0.00567 ± 0.000717	0.00415–0.00674	89.4 (4)
	Elastic Net	0.00594 ± 0.000897	0.0044–0.00806	0.00614 ± 0.000821	0.00461–0.00811	82.8 (5)
(E,E)-3,5-octadien-2-one	Random Forest	0.128 ± 0.0512	0.0394–0.242	0.151 ± 0.057	0.0428–0.271	3.59 (1)
	Bayesian Ridge	0.138 ± 0.0634	0.0347–0.26	0.165 ± 0.0655	0.0393–0.266	3.31 (2)
	SVR	0.196 ± 0.04	0.137–0.293	0.206 ± 0.0411	0.154–0.306	2.49 (3)
	XGBoost	0.186 ± 0.116	0.0225–0.456	0.234 ± 0.134	0.0283–0.502	2.38 (4)
	Elastic Net	0.311 ± 0.0658	0.188–0.428	0.328 ± 0.0603	0.211–0.451	1.57 (5)
1-Octanol, 3,7-dimethyl-	Random Forest	0.176 ± 0.0616	0.0707–0.294	0.204 ± 0.0686	0.0767–0.326	2.63 (1)
	SVR	0.182 ± 0.0721	0.0549–0.318	0.214 ± 0.0779	0.0624–0.33	2.53 (2)
	Bayesian Ridge	0.22 ± 0.0776	0.0578–0.388	0.254 ± 0.0753	0.0717–0.4	2.11 (3)
	XGBoost	0.242 ± 0.0885	0.0885–0.4	0.28 ± 0.0954	0.108–0.417	1.91 (4)
	Elastic Net	0.296 ± 0.0814	0.138–0.442	0.334 ± 0.0796	0.152–0.476	1.59 (5)
2(3H)-Furanone, 5-heptyldihydro-	Random Forest	0.00834 ± 0.00319	0.00246–0.0134	0.00991 ± 0.00366	0.00287–0.0151	54.8 (1)
	XGBoost	0.00985 ± 0.00364	0.00473–0.0196	0.011 ± 0.00356	0.00584–0.0217	48 (2)
	Bayesian Ridge	0.0106 ± 0.00439	0.00323–0.0202	0.0126 ± 0.00492	0.00414–0.0226	43.1 (3)
	SVR	0.0142 ± 0.00439	0.00623–0.022	0.0161 ± 0.00479	0.00667–0.024	33 (4)
	Elastic Net	0.0147 ± 0.00388	0.00874–0.0215	0.0163 ± 0.00414	0.00878–0.0224	32.3 (5)

Phenotype	Model	MAE, mean ± SD	MAE empirical interval	RMSE, mean ± SD	RMSE empirical interval	Performance score (rank)
2(3H)-Furanone, 5-hexyldihydro-	Random Forest	0.0106 ± 0.00363	0.00428–0.0174	0.0121 ± 0.00355	0.00586–0.0183	44.1 (1)
	XGBoost	0.0129 ± 0.00482	0.00597–0.0235	0.0141 ± 0.00519	0.0064–0.0247	37.1 (2)
	Bayesian Ridge	0.0144 ± 0.00413	0.00722–0.0218	0.0162 ± 0.00403	0.00933–0.0235	32.7 (3)
	SVR	0.0169 ± 0.00583	0.00694–0.028	0.0194 ± 0.0056	0.00815–0.0292	27.5 (4)
	Elastic Net	0.0176 ± 0.00548	0.00793–0.0269	0.02 ± 0.00522	0.00974–0.0279	26.6 (5)
2-Cyclopenten-1-one, 2-hydroxy-3,4-dimethyl-	Random Forest	0.00751 ± 0.00254	0.00207–0.0113	0.0086 ± 0.00263	0.00277–0.0129	62.1 (1)
	Bayesian Ridge	0.00871 ± 0.00316	0.00366–0.0162	0.01 ± 0.00337	0.00409–0.0176	53.4 (2)
	XGBoost	0.0104 ± 0.00344	0.00359–0.0163	0.012 ± 0.00372	0.00458–0.0179	44.7 (3)
	SVR	0.0116 ± 0.00313	0.00624–0.018	0.0128 ± 0.00289	0.00666–0.0182	40.9 (4)
	Elastic Net	0.0122 ± 0.00332	0.00594–0.0183	0.0136 ± 0.00322	0.00625–0.0187	38.7 (5)
2-methoxy-Phenol	Random Forest	0.0556 ± 0.0254	0.0228–0.112	0.066 ± 0.0314	0.024–0.132	8.22 (1)
	Bayesian Ridge	0.0592 ± 0.0284	0.0142–0.122	0.0721 ± 0.0317	0.016–0.133	7.62 (2)
	XGBoost	0.0897 ± 0.0415	0.0157–0.182	0.114 ± 0.0443	0.021–0.201	4.91 (3)
	SVR	0.102 ± 0.0307	0.054–0.168	0.111 ± 0.0309	0.065–0.176	4.7 (4)
	Elastic Net	0.128 ± 0.0327	0.0671–0.186	0.136 ± 0.0329	0.0819–0.202	3.8 (5)
2-Propenoic acid, butyl ester	Random Forest	0.0217 ± 0.0097	0.00524–0.0398	0.0253 ± 0.0104	0.0075–0.0435	21.2 (1)
	Bayesian Ridge	0.0267 ± 0.0113	0.00689–0.053	0.0323 ± 0.0121	0.00947–0.0577	17 (2)
	XGBoost	0.0281 ± 0.0116	0.00833–0.0533	0.0331 ± 0.0128	0.00918–0.0559	16.3 (3)
	Elastic Net	0.0288 ± 0.0109	0.0102–0.0499	0.0329 ± 0.0111	0.0134–0.0539	16.2 (4)
	SVR	0.034 ± 0.00713	0.0212–0.0456	0.036 ± 0.00703	0.0224–0.0464	14.3 (5)
2-Propenoic acid, oxiranylmethyl ester	Random Forest	0.0192 ± 0.00729	0.006–0.0331	0.022 ± 0.00707	0.00751–0.0375	24.3 (1)
	Elastic Net	0.0214 ± 0.00798	0.00909–0.0369	0.0242 ± 0.00853	0.011–0.0407	21.9 (2)
	XGBoost	0.0216 ± 0.00811	0.00859–0.0409	0.0248 ± 0.0085	0.0106–0.0427	21.5 (3)
	Bayesian Ridge	0.0219 ± 0.00719	0.0113–0.0355	0.0253 ± 0.00717	0.0132–0.0432	21.2 (4)
	SVR	0.0229 ± 0.00816	0.0108–0.0389	0.0254 ± 0.00877	0.0126–0.0416	20.7 (5)

Phenotype	Model	MAE, mean ± SD	MAE empirical interval	RMSE, mean ± SD	RMSE empirical interval	Performance score (rank)
2-Undecanol	Random Forest	0.00852 ± 0.00349	0.00266–0.014	0.01 ± 0.00395	0.0031–0.0165	53.9 (1)
	XGBoost	0.011 ± 0.00508	0.00323–0.0237	0.0127 ± 0.0055	0.00372–0.0258	42.1 (2)
	Bayesian Ridge	0.0115 ± 0.00357	0.0057–0.018	0.0128 ± 0.0035	0.0075–0.019	41.1 (3)
	SVR	0.0152 ± 0.00498	0.00581–0.0238	0.0174 ± 0.00508	0.00748–0.0255	30.7 (4)
	Elastic Net	0.0157 ± 0.00443	0.00731–0.0229	0.0176 ± 0.00469	0.00844–0.0245	30 (5)
3,3-dimethyl-2-Hexanone	Random Forest	0.00999 ± 0.00375	0.00321–0.0165	0.0111 ± 0.00388	0.00345–0.0179	47.3 (1)
	Elastic Net	0.0118 ± 0.00461	0.00364–0.0208	0.0136 ± 0.00473	0.00451–0.0223	39.5 (2)
	Bayesian Ridge	0.0118 ± 0.00431	0.0041–0.0218	0.0137 ± 0.00462	0.00481–0.0242	39.1 (3)
	XGBoost	0.012 ± 0.00496	0.00369–0.0226	0.0141 ± 0.00536	0.00445–0.023	38.3 (4)
	SVR	0.0133 ± 0.00337	0.00765–0.0193	0.0142 ± 0.00337	0.00808–0.0196	36.4 (5)
3-Cyclohexen-1-ol, 1-methyl-4-(1-methylethyl)-	XGBoost	0.00317 ± 0.0015	0.00129–0.00634	0.00354 ± 0.0016	0.0014–0.00689	149 (1)
	Random Forest	0.00327 ± 0.00119	0.0014–0.00592	0.00367 ± 0.00126	0.00152–0.00629	144 (2)
	Elastic Net	0.0037 ± 0.00128	0.00136–0.00651	0.00418 ± 0.00132	0.00156–0.0068	127 (3)
	SVR	0.0041 ± 0.00103	0.00228–0.00599	0.00441 ± 0.00102	0.00252–0.00607	117 (4)
	Bayesian Ridge	0.00427 ± 0.00151	0.00144–0.00729	0.00513 ± 0.00168	0.00159–0.00826	106 (5)
6-Undecanol	Random Forest	0.00293 ± 0.00122	0.00106–0.0054	0.0035 ± 0.00152	0.00119–0.00619	156 (1)
	XGBoost	0.00362 ± 0.00124	0.00169–0.00621	0.00422 ± 0.00141	0.00171–0.00655	128 (2)
	Bayesian Ridge	0.00403 ± 0.00118	0.0021–0.00596	0.0046 ± 0.0013	0.00247–0.00676	116 (3)
	Elastic Net	0.00446 ± 0.00163	0.00156–0.00779	0.0052 ± 0.00188	0.00187–0.0081	104 (4)
	SVR	0.00468 ± 0.00173	0.00159–0.00781	0.00553 ± 0.00205	0.00187–0.00848	97.9 (5)
b*45min	SVR	0.724 ± 0.252	0.265–1.23	0.828 ± 0.269	0.325–1.3	0.644 (1)
	Random Forest	0.774 ± 0.265	0.32–1.3	0.894 ± 0.302	0.39–1.41	0.6 (2)
	Elastic Net	0.82 ± 0.198	0.414–1.19	0.89 ± 0.192	0.474–1.22	0.585 (3)
	XGBoost	0.789 ± 0.319	0.255–1.42	0.932 ± 0.37	0.305–1.53	0.581 (4)
	Bayesian Ridge	0.803 ± 0.284	0.302–1.44	0.932 ± 0.317	0.38–1.6	0.576 (5)

Phenotype	Model	MAE, mean ± SD	MAE empirical interval	RMSE, mean ± SD	RMSE empirical interval	Performance score (rank)
Benzene, (1,1-dimethylethoxy)-	Random Forest	0.00313 ± 0.00078	0.00188–0.00445	0.00334 ± 0.000929	0.00189–0.00503	155 (1)
	XGBoost	0.00321 ± 0.0012	0.000987–0.0055	0.00398 ± 0.00139	0.00116–0.00585	139 (2)
	Bayesian Ridge	0.00347 ± 0.00118	0.00169–0.00645	0.00386 ± 0.00141	0.00188–0.00722	137 (3)
	SVR	0.00435 ± 0.00107	0.00258–0.00591	0.0047 ± 0.00088	0.00325–0.00591	110 (4)
	Elastic Net	0.00465 ± 0.0012	0.00265–0.00624	0.0051 ± 0.00102	0.00341–0.00646	103 (5)
Butanoic acid, 3-methyl-, 3-methylbutyl ester	Random Forest	0.0251 ± 0.00866	0.0112–0.0406	0.0285 ± 0.0102	0.0129–0.0475	18.6 (1)
	Bayesian Ridge	0.0293 ± 0.00967	0.0101–0.0496	0.0333 ± 0.00989	0.0119–0.0555	16 (2)
	XGBoost	0.0324 ± 0.0128	0.00854–0.0547	0.0372 ± 0.0143	0.00916–0.0573	14.4 (3)
	SVR	0.041 ± 0.0114	0.0173–0.0612	0.0456 ± 0.0108	0.0223–0.0642	11.5 (4)
	Elastic Net	0.041 ± 0.0111	0.0182–0.063	0.0457 ± 0.0113	0.0194–0.0672	11.5 (5)
Butyl propanoate	Random Forest	0.0163 ± 0.00626	0.00707–0.0285	0.0182 ± 0.0066	0.00754–0.0312	29 (1)
	Elastic Net	0.0192 ± 0.00768	0.00699–0.0342	0.0223 ± 0.00786	0.00867–0.0365	24.1 (2)
	Bayesian Ridge	0.0197 ± 0.00729	0.00766–0.0366	0.0231 ± 0.00773	0.00919–0.0407	23.4 (3)
	SVR	0.0219 ± 0.00549	0.0138–0.0316	0.0235 ± 0.00555	0.0148–0.0322	22 (4)
	XGBoost	0.0214 ± 0.00801	0.00763–0.0365	0.0245 ± 0.00862	0.00811–0.0385	21.8 (5)
CH3C(O)O(CH2)3CH=CH2	Random Forest	0.012 ± 0.0054	0.0041–0.0228	0.0139 ± 0.00616	0.00431–0.0259	38.6 (1)
	Bayesian Ridge	0.0146 ± 0.00611	0.00504–0.0288	0.0176 ± 0.00669	0.00558–0.032	31.1 (2)
	Elastic Net	0.0156 ± 0.00597	0.00666–0.0277	0.0179 ± 0.0064	0.00781–0.0303	29.8 (3)
	XGBoost	0.0168 ± 0.00703	0.00483–0.0308	0.0198 ± 0.008	0.0068–0.0331	27.4 (4)
	SVR	0.0186 ± 0.00416	0.0122–0.0257	0.0198 ± 0.00434	0.0126–0.0268	26 (5)
Circumference of muscle fiber	SVR	13 ± 7.56	3.2–27.9	17.1 ± 9.84	4.23–32.8	0.0333 (1)
	Random Forest	14.5 ± 5.58	5.85–27	17.3 ± 6.77	6.44–31	0.0315 (2)
	Elastic Net	15.7 ± 6.48	4.12–27.3	18.7 ± 7.44	4.95–31.3	0.029 (3)
	Bayesian Ridge	16.5 ± 6.26	6.75–29	19.5 ± 7.09	7.35–33.5	0.0278 (4)
	XGBoost	20.9 ± 7.38	6.56–33.6	24.4 ± 7.87	6.69–36.8	0.0221 (5)

Phenotype	Model	MAE, mean ± SD	MAE empirical interval	RMSE, mean ± SD	RMSE empirical interval	Performance score (rank)
Cyclohexanol, 1-methyl-4-(1-methylethenyl)-	XGBoost	0.00317 ± 0.0015	0.00129–0.00634	0.00354 ± 0.0016	0.0014–0.00689	149 (1)
	Random Forest	0.00327 ± 0.00119	0.0014–0.00592	0.00367 ± 0.00126	0.00152–0.00629	144 (2)
	Elastic Net	0.0037 ± 0.00128	0.00136–0.00651	0.00418 ± 0.00132	0.00156–0.0068	127 (3)
	SVR	0.0041 ± 0.00103	0.00228–0.00599	0.00441 ± 0.00102	0.00252–0.00607	117 (4)
	Bayesian Ridge	0.00427 ± 0.00151	0.00144–0.00729	0.00513 ± 0.00168	0.00159–0.00826	106 (5)
Ethanone, 1-(4,5-dihydro-2-thiazolyl)-	Random Forest	0.00577 ± 0.00235	0.00234–0.0102	0.00664 ± 0.00266	0.00286–0.0113	80.6 (1)
	Bayesian Ridge	0.00721 ± 0.00283	0.00142–0.012	0.00828 ± 0.00305	0.00159–0.0129	64.6 (2)
	XGBoost	0.00749 ± 0.00323	0.00181–0.0152	0.0089 ± 0.00331	0.00209–0.0156	61 (3)
	Elastic Net	0.0089 ± 0.00324	0.0028–0.0142	0.0101 ± 0.00348	0.00364–0.0155	52.6 (4)
	SVR	0.00903 ± 0.00327	0.00311–0.0139	0.0106 ± 0.00356	0.00332–0.015	50.9 (5)
Glutaraldehyde	Random Forest	0.0134 ± 0.00556	0.00398–0.0237	0.0153 ± 0.00593	0.00474–0.0257	34.8 (1)
	Bayesian Ridge	0.0161 ± 0.00641	0.00551–0.0309	0.0192 ± 0.00695	0.00606–0.0338	28.3 (2)
	Elastic Net	0.0175 ± 0.00683	0.00547–0.0306	0.0201 ± 0.00693	0.00717–0.033	26.6 (3)
	XGBoost	0.0179 ± 0.00732	0.00565–0.0341	0.0211 ± 0.00798	0.00724–0.0345	25.6 (4)
	SVR	0.0199 ± 0.00489	0.012–0.0282	0.0213 ± 0.00484	0.0128–0.0288	24.2 (5)
Gumminess	SVR	0.67 ± 0.267	0.202–1.27	0.785 ± 0.285	0.213–1.37	0.688 (1)
	Elastic Net	0.68 ± 0.257	0.331–1.27	0.783 ± 0.287	0.385–1.35	0.684 (2)
	Random Forest	0.719 ± 0.219	0.307–1.18	0.812 ± 0.232	0.388–1.3	0.653 (3)
	XGBoost	0.806 ± 0.274	0.294–1.37	0.923 ± 0.281	0.333–1.51	0.578 (4)
	Bayesian Ridge	0.944 ± 0.23	0.542–1.38	1.06 ± 0.246	0.636–1.45	0.499 (5)
L*24h	SVR	1.48 ± 0.597	0.518–2.63	1.73 ± 0.642	0.59–2.84	0.312 (1)
	Random Forest	1.54 ± 0.671	0.449–2.76	1.82 ± 0.748	0.513–2.89	0.298 (2)
	XGBoost	1.65 ± 0.855	0.238–3.33	1.87 ± 0.904	0.291–3.49	0.284 (3)
	Elastic Net	1.74 ± 0.55	0.836–2.69	2 ± 0.605	0.934–2.85	0.267 (4)
	Bayesian Ridge	1.87 ± 0.679	0.625–3.31	2.14 ± 0.726	0.828–3.46	0.249 (5)

Phenotype	Model	MAE, mean ± SD	MAE empirical interval	RMSE, mean ± SD	RMSE empirical interval	Performance score (rank)
L*45min	Random Forest	2.76 ± 0.879	1.44–4.54	3.06 ± 1.02	1.47–4.93	0.172 (1)
	SVR	2.76 ± 0.948	1.13–4.43	3.11 ± 0.997	1.24–4.66	0.17 (2)
	Elastic Net	2.88 ± 1	1.48–4.88	3.14 ± 1.12	1.71–5.34	0.166 (3)
	Bayesian Ridge	3.33 ± 0.895	2.07–5.57	3.59 ± 1.04	2.16–6.43	0.145 (4)
	XGBoost	3.26 ± 1.16	1.27–5.93	3.71 ± 1.23	1.65–6.64	0.143 (5)
Muscle CAT	Random Forest	7.33 ± 3.99	2.21–14.2	9.21 ± 5.5	2.52–18.1	0.0605 (1)
	Bayesian Ridge	8.25 ± 4.77	2.21–19.9	10.2 ± 6.23	2.54–23.6	0.0542 (2)
	Elastic Net	9.78 ± 5.17	1.96–19.8	12.3 ± 6.3	2.32–23.2	0.0454 (3)
	SVR	10.2 ± 4.74	4.12–20.7	12.1 ± 6.27	4.64–23.8	0.0448 (4)
	XGBoost	10.7 ± 5.69	1.07–20.9	14.4 ± 7.55	1.35–25.2	0.0398 (5)
Muscle fiber cross-sectional area	SVR	287 ± 189	63–661	389 ± 269	71.3–813	0.00148 (1)
	Random Forest	319 ± 135	107–606	399 ± 177	113–725	0.00139 (2)
	Bayesian Ridge	355 ± 151	128–685	435 ± 185	156–821	0.00127 (3)
	Elastic Net	386 ± 155	112–698	467 ± 187	119–818	0.00117 (4)
	XGBoost	452 ± 184	62.3–757	569 ± 203	69–840	0.000979 (5)
Muscle GSH-Px	Random Forest	80.3 ± 48.7	10.5–193	103 ± 59.2	14.1–213	0.00545 (1)
	Bayesian Ridge	86.9 ± 48.9	15.9–223	113 ± 61.3	18.3–255	0.00501 (2)
	Elastic Net	92.3 ± 50.6	20.1–216	119 ± 63.4	25.1–246	0.00473 (3)
	SVR	95.9 ± 56.3	16.5–238	130 ± 73.1	18.5–264	0.00443 (4)
	XGBoost	118 ± 53.4	21.5–199	151 ± 65.5	23.2–230	0.00371 (5)
Muscle perimysium thickness	Bayesian Ridge	0.112 ± 0.0474	0.0258–0.2	0.136 ± 0.0523	0.0267–0.216	4.03 (1)
	Random Forest	0.195 ± 0.0765	0.0811–0.342	0.223 ± 0.0769	0.095–0.356	2.39 (2)
	XGBoost	0.283 ± 0.164	0.0711–0.71	0.333 ± 0.179	0.0951–0.792	1.62 (3)
	SVR	0.293 ± 0.144	0.0729–0.559	0.342 ± 0.148	0.0896–0.618	1.58 (4)
	Elastic Net	0.545 ± 0.142	0.302–0.802	0.57 ± 0.136	0.332–0.81	0.897 (5)

Phenotype	Model	MAE, mean ± SD	MAE empirical interval	RMSE, mean ± SD	RMSE empirical interval	Performance score (rank)
Springiness	Bayesian Ridge	0.195 ± 0.0677	0.0761–0.336	0.224 ± 0.071	0.0796–0.356	2.39 (1)
	Random Forest	0.217 ± 0.0686	0.0968–0.356	0.245 ± 0.0653	0.126–0.364	2.16 (2)
	SVR	0.233 ± 0.0783	0.122–0.394	0.258 ± 0.09	0.122–0.42	2.04 (3)
	XGBoost	0.278 ± 0.108	0.0993–0.46	0.315 ± 0.114	0.127–0.51	1.69 (4)
	Elastic Net	0.281 ± 0.0946	0.132–0.489	0.324 ± 0.092	0.182–0.5	1.65 (5)

Note. Values for MAE and RMSE are presented as mean ± SD. Empirical intervals represent the 2.5th to 97.5th percentiles across 100 repeated 80/20 train/test splits. Models were ranked separately within each phenotype according to the performance score, with rank 1 representing the best-performing model.
